# Transcriptional programs controlling sporulation in *Phytophthora* respond to nutrition, humidity, and cryptochrome-mediated light signals

**DOI:** 10.1371/journal.ppat.1014705

**Published:** 2026-10-12

**Authors:** Andrea L. Vu, Timothy A. Harris, Howard S. Judelson

**Affiliations:** Department of Microbiology and Plant Pathology, University of California, Riverside, United States of America; Utrecht University Faculty of Science: Universiteit Utrecht Faculteit Betawetenschappen, NETHERLANDS, KINGDOM OF THE

## Abstract

Many filamentous pathogens employ environmental signals to regulate sporulation, maximizing the likelihood that their spores survive to colonize a new host. In the oomycete *Phytophthora,* little is known of the transcriptional program that underlies sporangia formation and how it is influenced by environmental inputs such as light, humidity, and nutrients. By studying time courses of the potato blight pathogen *P. infestans* on artificial media and tomato leaves*,* we identified over 4000 genes up-regulated during sporulation. These are associated with signal transduction, cell structure, vesicle trafficking, metabolism, flagellar assembly, and other processes required for sporangium formation and germination. Most of their mRNAs remain abundant in mature sporangia. Media manipulation and metabolomic studies indicated that sporulation is triggered by nitrogen limitation, with asparagine and possibly other amino acids playing the main role. Light, which delays sporulation in *P. infestans,* apparently influences a later regulatory step since while many sporulation-induced transcripts oscillate with a day/night cycle, others are light-neutral. Both blue and red wavelengths result in the light response, which is likely mediated by a cryptochrome. One cryptochrome was shown to be required for sporulation based on gene silencing experiments. Humidity appears to regulate a late checkpoint, since while spore development requires high humidity, low humidity has only limited effects on sporulation-associated transcription. Comparative studies with *P. capsici,* which unlike *P. infestans* requires light for sporulation, yielded evidence of substantial rewiring of the transcriptional program between the species. The contrasting light responses within the genus correlate with whether species are primarily leaf colonizers such as *P. infestans* or root colonizers such as *P. capsici.*

## Author summary

Many disease-causing microbes use environmental cues to control the production of spores, which are used to spread the pathogen to new hosts. To better understand sporulation, we tracked changes in gene activity during spore production by *Phytophthora infestans*, a major pathogen of potato and tomato. We identified several thousand genes that become active during sporulation to build spores and prepare them for germination. We found that a shortage of amino acids triggers spore formation, and that the pathways that control sporulation respond to light and humidity. Species that primarily infect leaves, such as *P. infestans*, mostly sporulate at night, which minimizes damage to their spores from daytime light and heat. In contrast, light stimulates spore production in species of *Phytophthora* that colonize underground plant tissues, promoting sporulation on tissues emerging from soil where dispersal is more likely. These contrasting responses to light were associated with species-specific changes in the transcriptional program that controls sporulation. Our findings identify biological pathways that could be targeted by new chemicals to help reduce crop losses caused by disease.

## Introduction

Many microbial pathogens use environmental cues to regulate growth, development, and host colonization. Examples include the fungus *Ustilago,* which senses plant phenolics to transition to pathogenic growth [1], animal-infecting bacteria that activate their virulence genes when they detect body temperatures [2], bacteria and fungi that use nutrient limitation to trigger sporulation [3,4], fungi for which humidity influences sporulation [5], and fungi for which light dictates sporulation or directional growth [6]. Responses to these cues are not monolithic and may vary with the life strategy of the host. For example, light commonly stimulates spore production in fungi that spread using dry airborne spores, which is logical since daytime light contributes to their desiccation [4]. In contrast, light suppresses sporulation in many fungi that form hydrated spores that would suffer from drying [7]. Although proteins that detect environmental signals have been identified in several systems, how they regulate development is known only in limited cases [1,4,6,8,9].

Oomycetes also respond to environmental cues. These filamentous eukaryotic microbes belong to the stramenopile lineage, which also includes diatoms and brown algae [10]. Although oomycetes and fungi evolved independently, both occupy similar ecological niches, typically exhibit filamentous growth, and commonly use asexual spores for dissemination. That oomycete sporulation is sensitive to the environment is documented for several species in the plant pathogenic genus *Phytophthora* and related downy mildews. For example, sporulation usually requires high relative humidity (>95% RH), although some species may sporulate more at lower humidity levels [11–13]. Varying between species is whether light inhibits, stimulates, or has neutral effects on sporulation. For example, light suppresses sporulation in *Phytophthora infestans* [14,15] but is essential for sporulation in *P. capsici* [16]*.* Nutrient limitation has been suggested to regulate sporulation, but this is not well understood. In some species, sporangia develop only after nutrients are washed from the culture medium [16,17], while sporulation occurs readily without such treatment in others such as *P. infestans*.

Combined with its major economic importance as a pathogen of potato and tomato [18], the ability of *P. infestans* to form sporangia without media manipulations or light regimes has made it a model for studies of sporulation. This normally begins when cultures (or plant lesions) reach several days of age, but it is unknown if this is due to changes in the media (or host) or other factors leading to “sporulation competence” [19]. Sporulation begins with the formation of aerial hyphae that produce sporangiophore initials, which elongate into branched sporangiophores. Cytoplasm and organelles flow toward the branch tips, each of which swells to form a multinucleate sporangium. These steps take about eight hours to complete [20]. Unlike many fungi which delineate vegetative hyphae from spores by intermediate cell types, oomycete hyphae lack septa and there is continuity between the cytoplasm of the developing sporangium and vegetative hyphae. This continuity persists until a plug forms at the base of the sporangium as it matures [21]. In nature, wind or water flow will cause the sporangiophore to break at the plug, releasing the sporangium for dispersal to new hosts [22]. Depending on temperature, a sporangium will either emit a germ tube directly or partition its cytoplasm into flagellated zoospores [23]. Inhibitor studies indicated that all proteins required for zoosporogenesis are already present in the sporangium [24,25]. Sporangia are fully hydrated, metabolically active, and unlike many fungi lack pigments that might otherwise protect them from solar radiation. They consequently remain viable for shorter periods than most fungal spores [26,27].

The molecular genetics underlying sporangia formation in oomycetes remain poorly understood. Excluding genes with pleiotropic effects on development [28–30], only a handful of genes required for sporulation have been identified, including the MADS-box transcription factor and the Cdc14 protein phosphatase [31,32]. RNA-seq, microarray, and proteomic studies in *P. infestans* have catalogued differences in the mRNA and protein content of nonsporulating mycelia and mature sporangia [33–35]. However, remaining to be studied is the process of sporulation itself and how this is influenced by environment.

Our goal here was to fill these knowledge gaps*.* RNA-seq analysis of *P. infestans* growing on artificial media and tomato leaflets revealed major transcriptional changes during sporulation, with more than one-quarter of genes up-regulated. Media manipulation studies combined with metabolite profiling during growth in culture media and *in planta* supported the hypothesis that reduced levels of an amino acid such as asparagine are the primary triggers of sporulation. Light appeared to regulate a subsequent stage of sporulation since it did not affect all sporulation-induced genes, and gene silencing studies indicated that a cryptochrome regulated sporulation. Humidity strongly regulated sporulation but appeared to control a late developmental checkpoint since low humidity only blocked expression of a subset of sporulation-associated genes. Comparative studies with *P. capsici,* which unlike *P. infestans* requires light for sporulation, provided evidence for substantial rewiring of the transcriptional program underlying sporulation in the two species. The differences in their response to light relate to their respective lifestyles.

## Results

### Nitrogen limitation stimulates sporulation in *P. infestans*

A media manipulation study was performed to test the connection between nutrient depletion and asexual sporulation. This relationship was unclear since *P. infestans* normally sporulates after a few days of growth on culture media, in contrast with many other *Phytophthora* spp. for which substantial sporulation occurs only after nutrients are removed by buffer washes. To help investigate this*,* we developed a protocol in which an inoculum of sporangia is spread on a polycarbonate membrane (0.4 µm pore size) resting on rye-sucrose agar. The membrane allows the growing hyphal mat to be transferred to other conditions without disruption, and aids recovery of the tissue for subsequent analyses. When maintained on rye-sucrose media, new sporangia typically begin to appear after about four days, with their numbers plateauing over the next two to three days.

Fig 1A shows the use of this procedure to test the effect of nitrogen and carbon depletion on sporulation. When the membrane was lifted and replaced on the now-used rye-sucrose agar after 72 h (pre-sporulation), sporangia began to be detected after 12 additional hours. Sporulation accelerated about ten-fold when the membrane was shifted to water agar or agar containing only glucose and sucrose. Transferring the filters to fresh rye-sucrose media delayed sporulation by about one day. Sporulation was also delayed by agar containing 1% casamino acids, an acid hydrolysate of casein. Thus, nitrogen limitation appears to be a more significant inducer of sporulation than carbon depletion.

**Fig 1:**
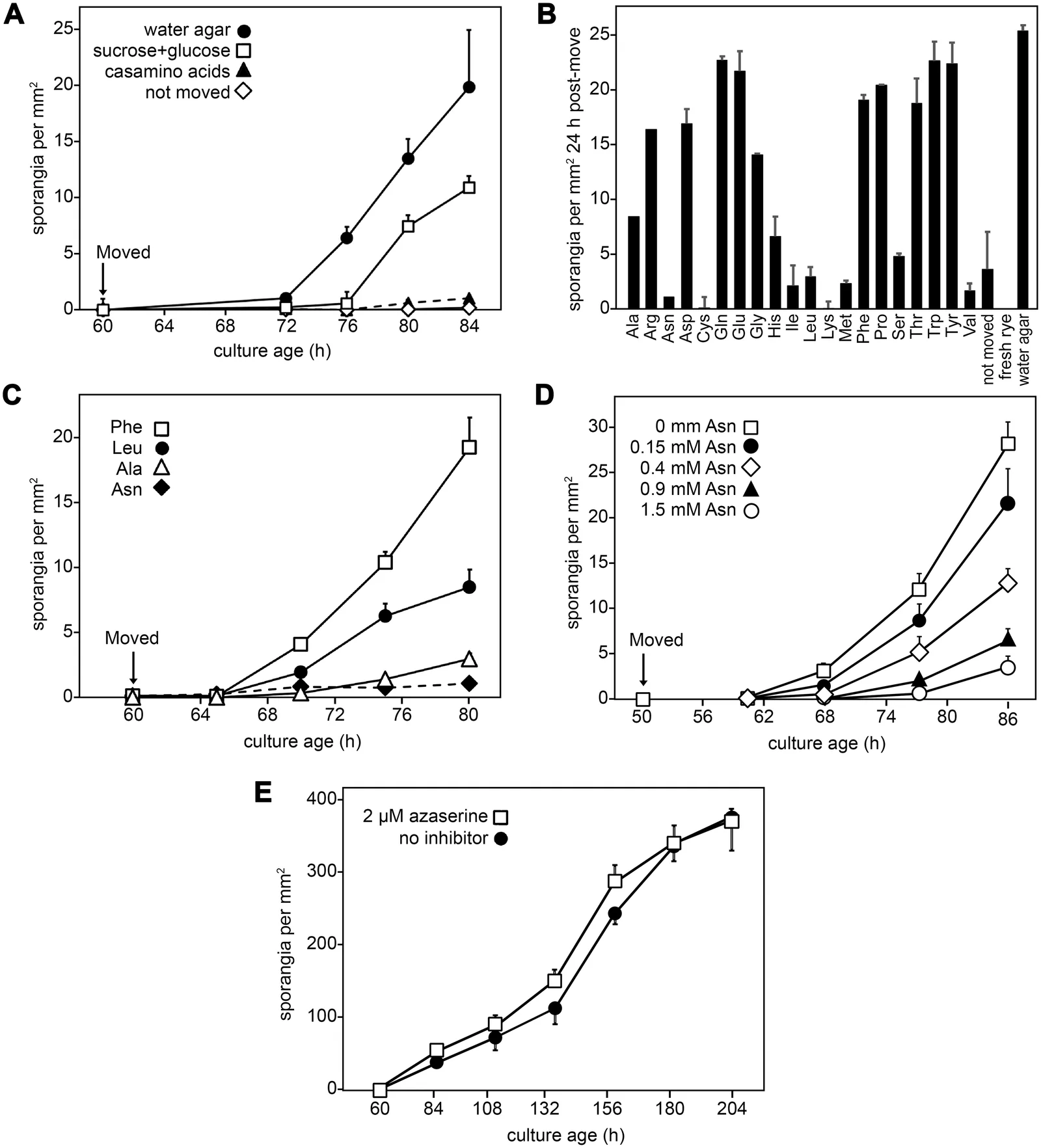
Amino acids suppress sporulation by *P. infestans.* **(A)** Sporulation by cultures grown on rye-sucrose agar which were shifted after 72 h to water agar, agar containing 10 mM sucrose and glucose, agar containing 1% casamino acids, fresh agar, or returned to the original media (labeled as used rye). **(B)** Ability of amino acids to suppress sporulation. After 48 h of growth, cultures were shifted to water agar containing 10 mM of the indicated amino acids, water agar alone, or fresh rye-sucrose media or returned to the original media. The experiment was performed using a time course, but only the 72 h timepoint is shown. Lysine and cysteine blocked sporulation, but this is believed due to toxicity. **(C)** Same experiment as in panel B but showing multiple timepoints for representative amino acids. **(D)** Same as panel C but with different concentrations of asparagine. **(E)** Influence of 2 µM azaserine on sporulation. Higher concentrations were also tested, but severely impaired growth.

This study and those described below were performed with three biological replicates per timepoint. They were also repeated at least twice in separate weeks, with similar conclusions. In different experiments the membranes were transferred at varying times after inoculation (48–72 h), as our intent was to equalize hyphal biomass at the time of transfer and batches of sporangia germinated at different rates. However, when conditions were being tested within an experiment all membranes were transferred at the same time. Several preliminary experiments indicated the timing of membrane transfer was a critical variable; sporulation levels were poor if transfer to water agar was too early, while no treatment was very effective at arresting sporulation if the transfer was too late.

Using the membrane transfer approach, we found that certain amino acids are more potent suppressors of sporulation than others. This involved growing cultures on rye-sucrose agar which were later lifted and transferred to water agar, fresh rye-sucrose media, or agar bearing 10 mM of each amino acid at pH 7.0. In addition, some membranes were replaced on the used media as a control. Sporulation was then measured every 4 h, and the results 24 h post-transfer are shown in Fig 1B. Consistent with the prior experiment, the transfer to water agar accelerated sporulation while fresh rye-sucrose media delayed sporulation. Several amino acids, especially asparagine and to a lesser extent valine, suppressed sporulation most strongly. About half of the amino acids, including phenylalanine and glutamate, either accelerated or failed to delay sporulation compared to the used media control. Others such as leucine and alanine had intermediate effects. These trends were consistent across multiple timepoints, as illustrated for representative amino acids in Fig 1C.

No sporulation occurred in cultures transferred to cysteine or lysine, but this appeared due to toxicity rather than a specific effect on sporulation. This is because when cultures were transferred to another condition (even water agar) hyphal biomass normally increased for at least 24 hours. However, growth stopped immediately when transfers were to water agar containing cysteine or lysine. This was not due to a pH effect since the amino acid treatments were done at pH 7.0.

The experiments in Fig 1B and 1C were performed with 10 mM of each amino acid, which is higher than would be found in most plant tissues. Prior studies measured asparagine at about 200 µM in tomato apoplasts and 1.3 mM in tomato fruit apoplasts and whole leaves [36,37]. We therefore repeated the asparagine treatment at concentrations ranging from 0.15 to 1. 5 mM. Each suppressed sporulation compared to plain water agar, although the effect was reduced at the lower concentrations and later timepoints (*i.e.,* 76 and 86 hr). Asparagine concentrations likely declined by the later timepoints, since the biomass of the hyphal mat continued to increase after the membrane transfer and since asparagine can serve as both a nitrogen and carbon source.

We also tested the effect of an inhibitor of asparagine synthetase, azaserine, on sporulation in rye-sucrose agar (Fig 1E). This increased the intensity of sporulation by about 20% until day 7, when sporulation with and without the inhibitor equalized, presumably due to amino acid depletion in both treatments. This experiment was performed at 2 µM azaserine, which reduced growth by about 20%. The results are consistent with a role of asparagine in regulating sporulation, but a caveat is that azaserine and other inhibitors of asparagine synthetase also affect purine synthesis, which could also affect sporulation [38].

### Declining amino acids are correlated with sporulation in *P. infestans*

The results presented in Fig 1A imply that amino acid depletion occurs near the onset of sporulation. We tested this in rye-sucrose agar cultures and infected tomato leaflets by extracting metabolites followed by high resolution liquid chromatography-mass spectrometry (Fig 2, S1 Table). Sporulation occurred primarily between days 4 and 5 in both rye-sucrose cultures and tomato (Fig 2A, B). For the agar cultures, this included analysis of both hyphae and media, allowing amino acids to be measured in both *P. infestans* tissue and the external environment. Unfortunately, it is not possible to measure amino acid levels in the infected plant tissue outside of hyphae.

**Fig 2:**
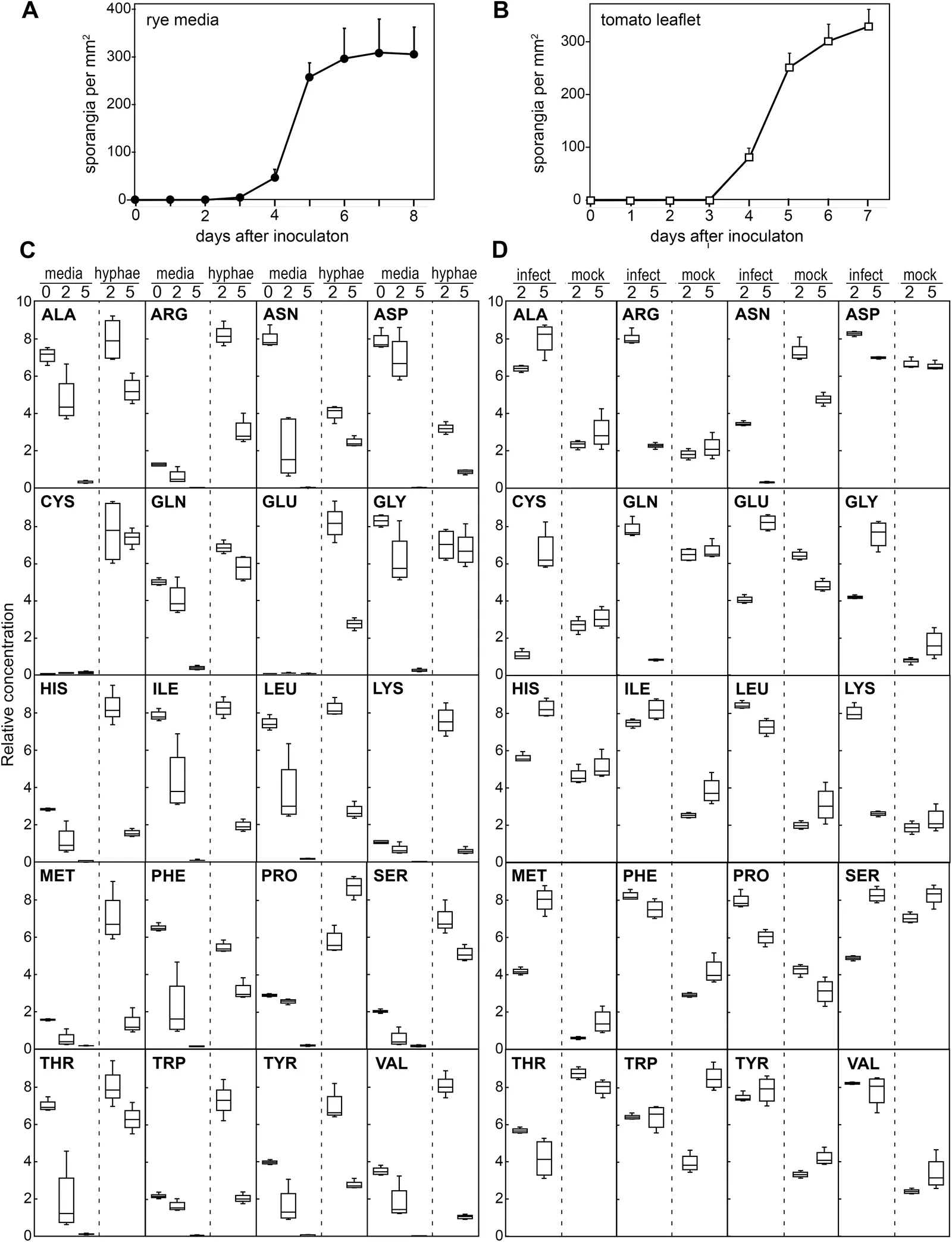
Timing of sporulation matches declines of amino acids in media and plant tissue. **(A)** Sporulation on rye-sucrose agar. **(B)** Sporulation on tomato leaflets. **(C)** Free amino acid levels during growth on rye-sucrose, indicating levels in hyphae at day 2 (pre-sporulation) and day 5 (post-sporulation) and in the media beneath the hyphae at days 0, 2, and 5. The values are relative and should not be compared between the different compounds. **(D)** Free amino acid levels in infected tomato leaflets and mock-infected controls, showing days 2 (pre-sporulation) and 5 (post-sporulation).

In the rye-sucrose cultures, most amino acids started to decline within the media by day 2 and fell precipitously by the time of sporulation on day 5 (Fig 2C). Since the analyses were performed without internal standards, the graphs illustrate relative levels of each amino acid. However, these can be converted to molarity using the content of the media shown in S2 Table. For example, asparagine declined from 289 µM in fresh media to 57 µM at day 2 and 0 µM at day 5. Strong declines were also seen for most other amino acids.

In hyphae from the same cultures, many amino acids also declined (Fig 2C). These changes were usually not as pronounced as those in media, and not all amino acids fell by the same amount. For example, amino acids such as asparagine and glutamate dropped in hyphae by 40% between day 2 and 5 and lysine fell by 92%, while little change was observed for glutamine and glycine.

Consistent with the persistence of sugars in the media, glucose levels showed little change in hyphae. However, decreases were observed in several glycolytic intermediates such as phosphoenolpyruvate, which might suggest increased glycolytic flux (S1A Fig). This may also explain why intermediates from the first part of the TCA cycle (citrate to isocitrate) rose upon sporulation (S1B Fig). In contrast, 3‑phosphoglycerate rose, suggesting either that phosphoglucomutase or enolase are rate-limiting steps in glycolysis or that there was less anabolic drain into serine through the phosphoserine aminotransferase pathway. The reduced levels of fumarate, malate, and oxaloacetate observed at day 5 might be explained by their use for producing aspartate, which is a precursor for several amino acids.

In infected tomato leaflets, concentrations of most amino acids were substantially different than mock-infected controls (Fig 2D). Nearly all showed little change or were higher at the day 5 sporulation timepoint compared to the day 2 pre-sporulation date. This was not surprising since increased amino acid levels is a feature of many plant defense responses, including during the infection of potato by *P. infestans* [39,40]. Only arginine, asparagine, glutamine, and lysine fell during infection, with the most extreme change being a 97% decline for asparagine and a 90% decline for glutamine. As the leaflets contained both tomato and *P. infestans* tissue, it is not possible to determine how the metabolites partitioned between host and pathogen. However, microscopic examination suggested that at least 80% of the biomass at day 5 was of plant origin although not all plant cells were intact. Measurements of *Phytophthora*-specific metabolites such as those in the taurine pathway suggested that between 5 and 20% of the metabolites were from the pathogen. Therefore, it seems that amino acids such as asparagine that showed precipitous declines were probably low both in *P. infestans* hyphae and the surrounding tomato tissue.

Although valine and asparagine had similar suppressive effects on sporulation in our filter transfer assays, only asparagine levels declined during tomato infection. It is therefore reasonable to consider that transporters or enzymes that act on asparagine might be sensors that regulate sporulation. Candidate enzymes include asparagine synthetases (genes PITG_13399 and PITG_16088), asparagine-tRNA ligase (PITG_05354 and PITG_12864), and asparaginase (PITG_10270). Interestingly, our *in vitro* assays indicated that the latter acts on asparagine and glutamine at similar rates, like some bacterial asparaginases (S2 Fig) [41]. Alternatively, since leucine and isoleucine also exhibited fairly strong suppressive effects, a sensor that detects branched-chain amino acids could also be involved, analogous to CodY of bacteria [42]. *P. infestans* lacks a CodY ortholog.

### Red and blue light repress sporulation in *P. infestans*

Previous studies documented the role of light in regulating sporulation in several oomycetes [14,16,43]. This includes our prior work where we showed that white light (throughout the paper, this refers to cool white fluorescent lamps) delayed sporulation by *P. infestans* and caused diurnal banding in cultures exposed to light/dark cycles [15]. To extend these findings and establish parameters for later experiments in this paper, we tested the effect of light wavelength and intensity on sporulation in *P. infestans.* This will be extended to *P. capsici* later in this paper.

As shown in Fig 3A, 75 µmole m^-2^ sec^-1^ of white light strongly repressed sporulation by *P. infestans.* The effect was dose-dependent (Fig 3B). Using light-emitting diode sources, blue and red were both found to have inhibitory effects, with blue being slightly more effective (Fig 3C). The latter contradicts a previous study performed *in planta* where only blue light had an effect [14]. A check of our red and blue light sources confirmed the purity of their outputs (Fig 3D).

**Fig 3:**
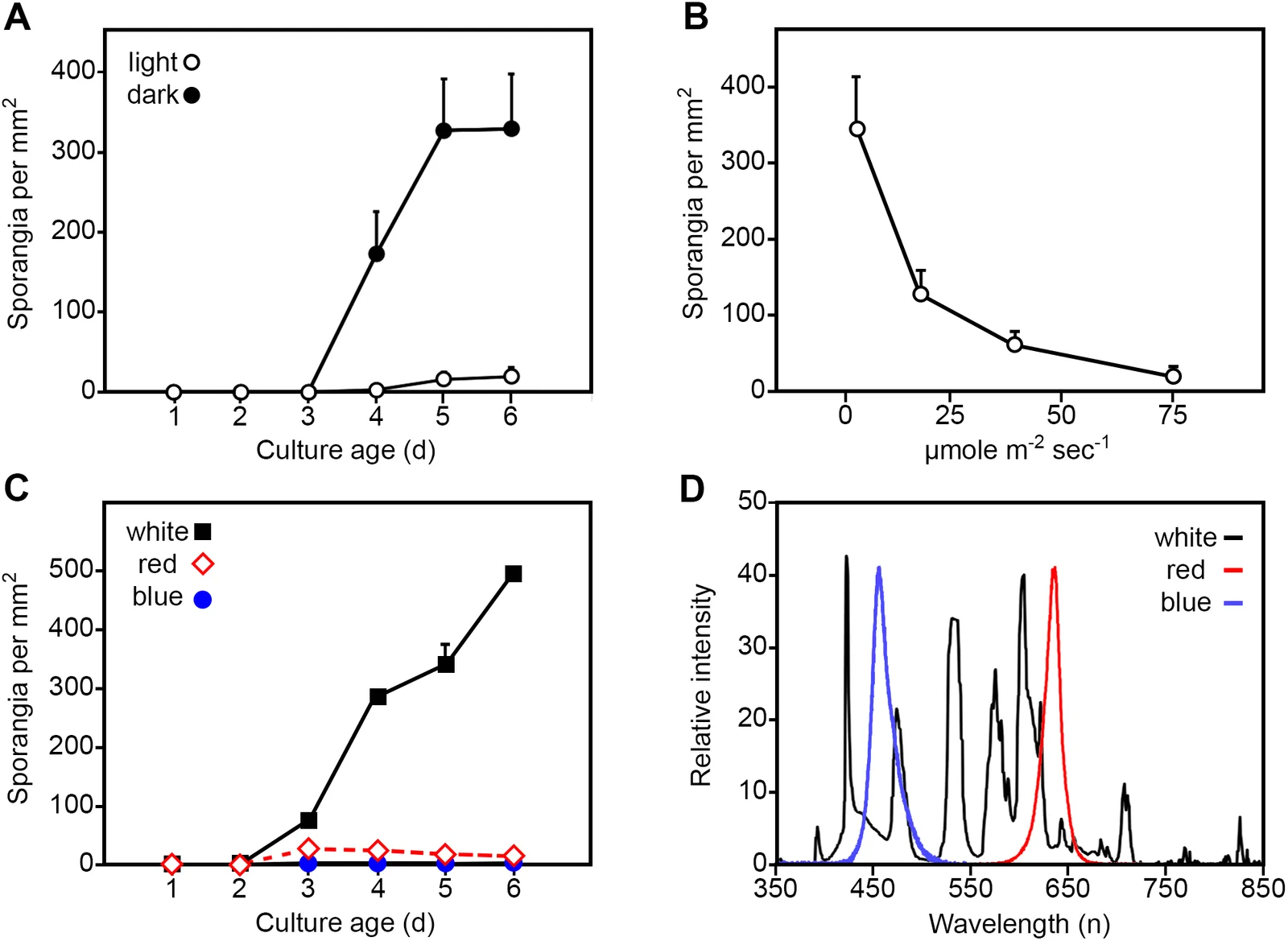
Blue and red light suppress sporulation by *P. infestans.* **(A)** Density of sporangia during a time course experiment under white light (75 µmole m^-2^ sec^-1^) or in darkness. **(B)** Density of sporangia in cultures exposed to varying amounts of white light, measured after seven days. **(C)** Effect of 75 µmole m^-2^ sec^-1^ white light, and 100 μmol m^-2^ sec^-1^ red or blue light on sporulation. **(D)** Spectral output of the white, red, and blue sources used in this study. Measurements are rescaled to have a similar maximum intensity in this panel.

### Massive changes in the *P. infestans* transcriptome occur during sporulation

We previously used RNA-seq to compare nonsporulating (young) mycelia and sporangia [34]. However, the sporulation process itself had not been examined, except for small-scale studies employing reverse transcription-polymerase chain reaction (RT-PCR) [15]. To fill this knowledge gap, we used RNA-seq to conduct a genome-wide survey using cultures grown on rye-sucrose media in constant darkness, constant light, and a 12 h light/dark cycle, and on tomato leaflets subjected to same light/dark regime S3 Table). Samples were taken every 12 h for the rye-sucrose media samples and every 4 h for the *in planta* experiment. The light/dark cycles used white lights at 32 µmole m^-2^ sec^-1^. Although our intent was not to mimic conditions within a potato field, this is similar to the intensity within the lower canopy where late blight most commonly develops [44,45]. As shown in S3 Fig, sporulation began around day 4 in each treatment and continued through days 7 or 8. However, sporulation proceeded faster and reached higher levels in the dark.

Heatmaps of the transcriptome during the time courses are shown in Fig 4A. Evident in each dataset is a large group of genes for which transcript levels increased dramatically during each time course. To estimate the number genes strongly induced during sporulation, we compared day 2 (pre-sporulation) with a sporulating timepoint. To adjust for variation in the pace of sporangia formation, we chose as the sporulating timepoint day 5.12 (5 days and 12 h, or 132 h after inoculation) for the dark rye-sucrose study, day 7.0 for the light rye-sucrose study, day 6.0 for the rye-sucrose study with the light/day cycle, and day 5.20 for the tomato experiment.

**Fig 4:**
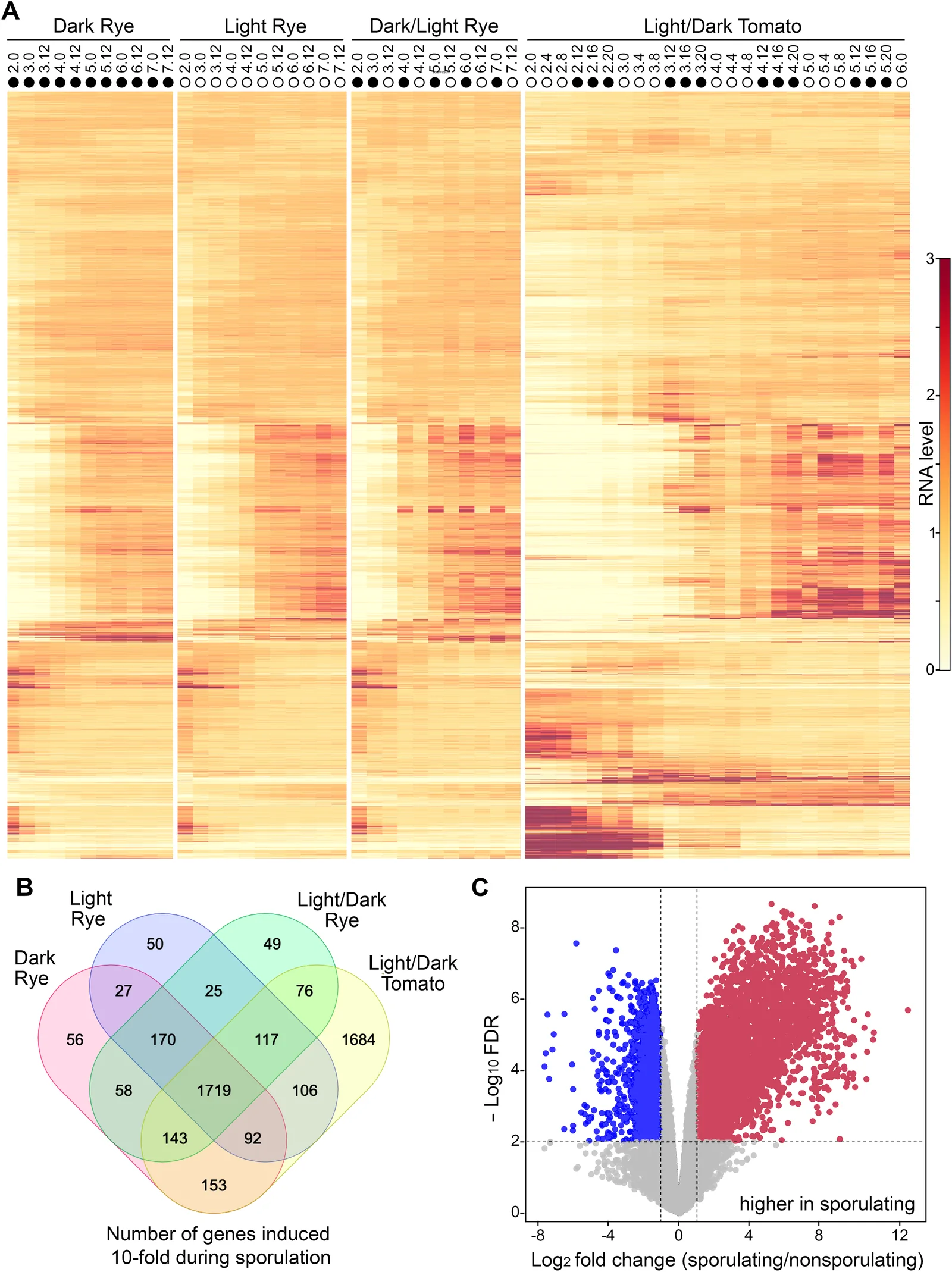
Massive changes in gene expression occur during sporulation. **(A)** mRNA levels during growth on rye-sucrose media in the dark, rye-sucrose media in the light, rye-sucrose media with a 12 h dark/light cycle, and tomato leaflets subjected to the light/dark cycle. In the light/dark time courses, timepoints with black and white circles represent samples taken at the end of the dark and light periods, respectively. Data are normalized such that each gene has a mean of 1.0 across the samples. **(B)** Overlap between number of genes expressed ^3^10-fold higher in the sporulation timepoints in each growth condition compared to the non-sporulating timepoint (day 2). **(C)** Volcano plot of mRNA levels at sporulating and non-sporulating timepoints based on edgeR analysis of the time courses.

We then used two approaches to assess the number of sporulation-induced genes. The first counted overlap between the genes induced by >10-fold in all four experiments. Out of 14,380 genes passing the low-count threshold, 1719 were induced by >10-fold (Fig 4B). A similar number was obtained using our second approach, which used edgeR and treated each time course as a biological replicate; the large number of data points made the collection of true biological replicates for each of the four time courses impractical. The edgeR results, which forms the basis for most subsequent analyses in this paper, identified 1832, 2936, and 4167 genes as up-regulated by >10-, 4-, and 2-fold, respectively, using an FDR cutoff of 0.05 (Fig 4C). Called as down-regulated by >10-, 4-, and 2-fold were 126, 552, and 2022 genes (S4 Table). Despite the lack of true biological replicates, the expression patterns of the up-regulated genes were consistent between the four time courses. However, some genes showing small changes in transcript levels may have been missed by this approach. Genes changing only in plants but not the three rye-sucrose experiments may also not have been called as differentially-expressed, although these are unlikely to be related to sporulation.

Due to the strong predominance of up-regulated genes, there was some concern that the normalization method had introduced bias. However, similar results were obtained when the default TMM normalization in edgeR was replaced with an adaptive method [46] that more aggressively excluded differentially-expressed genes from the normalization calculation. Also, comparative results were obtained using DESeq2 with its median-of-ratios normalization approach, which identified 1964 genes as >10-fold up-regulated compared to 1832 using edgeR. Similar values were obtained when normalization was based on housekeeping genes previously identified as suitable RT-qPCR controls [47].

Gene Ontology (GO) analysis indicated that most over-represented terms associated with the 1832 strongly up-regulated genes were related to flagella (cilia), ion channels, and protein kinases (Fig 5A). The latter included both catalytic domains and regulatory subunits that bind cAMP or cGMP. As shown in Fig 5B, over-represented GO terms associated with down-regulation were associated with protein synthesis (*e.g.,* ribosome, ribosome binding, translation factor) and metabolism (*e.g.,* oxidoreductase category). The latter is part of a much broader down-regulation of the metabolic transcriptome that will be described later.

**Fig 5:**
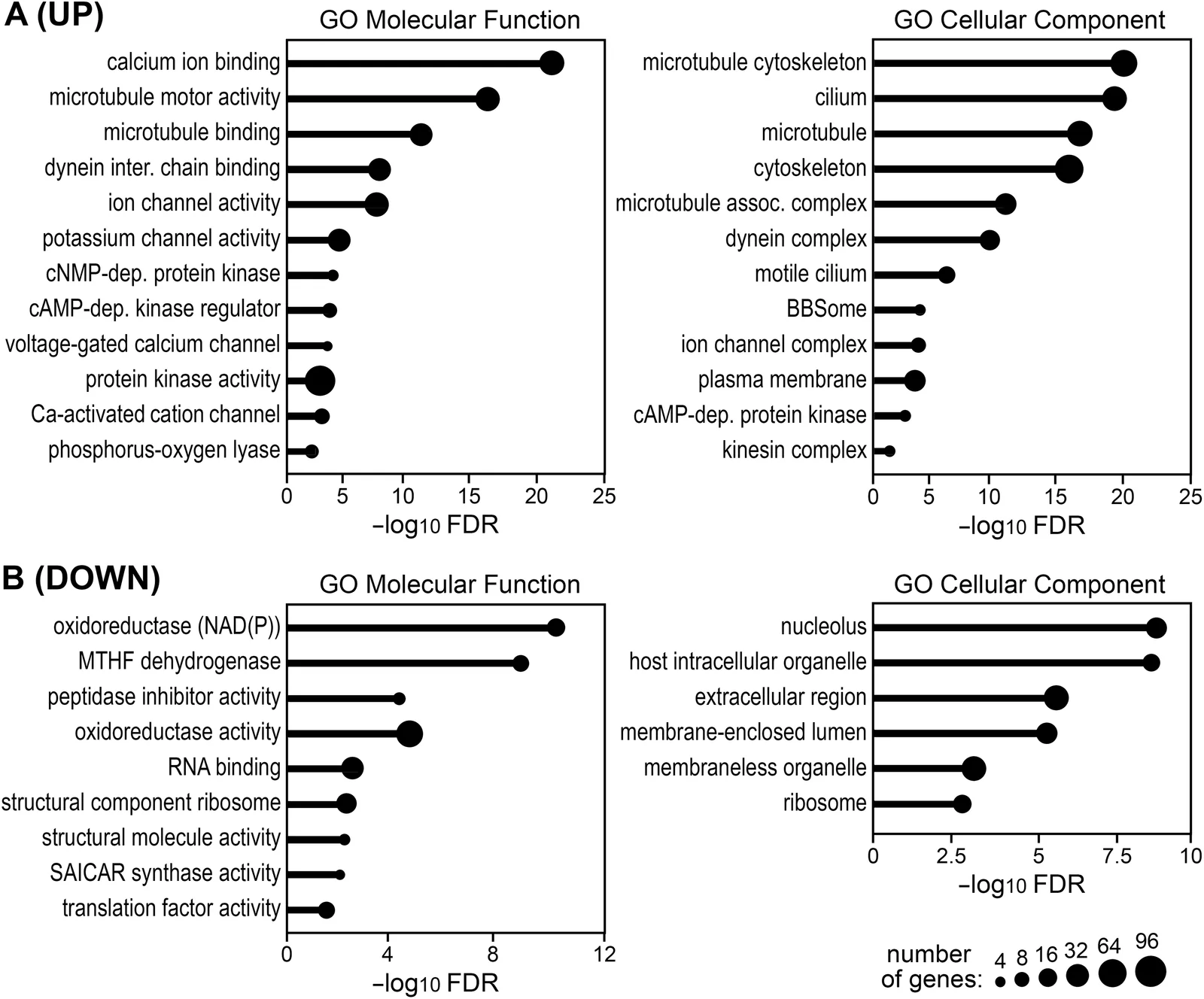
Several GO terms are enriched in genes differentially expressed during *P. infestans* sporulation. **(A)** The top over-represented Molecular Function and Cellular Component terms for genes up-regulated during sporulation. Note that terms labelled cilium apply to flagella as both have comparable structures [97]. **(B)** Top terms connected with down-regulated genes. The lists were simplified to reduce redundancy.

Outside of these over-represented groups were genes of diverse function. This is expected for a complex developmental process that involves altering hyphal morphology, forming new structures (sporangia), generating lipid and phosphagen energy reserves for sporangia, and other changes. For example, the up-regulated genes included those encoding proteins involved in vesicle trafficking such as syntaxins and RAS GTPases, non-flagellar cytoskeleton proteins, cell wall proteins such as transglutaminases, signaling proteins, transporters of lipids and other metabolites, and others.

### A minority of *P. infestans* genes induced in the time courses reflect aging

A complication of studying gene expression in *Phytophthora* is that sporulation occurs when cultures grow older. Therefore, genes up-regulated during time courses may encode proteins that make sporangia or those involved in aging. The latter may include adaptations to declining nutrients, responses to the waste products of metabolism, or the recycling of cellular components from older hyphae. Consequently, we tested two strategies for distinguishing “sporulation” from “aging” genes.

In our first approach, we examined *P. infestans* strains that we had silenced for the MADS-box transcription factor, which is required for sporulation [31]. The logic was that genes up-regulated during a time course in a non-sporulating MADS-minus strain will either be aging-related or act in sporulation prior to the MADS checkpoint. Using RNA-seq, we found that only 21% of the 1832 genes were induced two-fold or more in 6-day versus 2-day cultures of MADS mutants (Fig 6A, top). Thus, we conclude that at least 79% of the 1832 genes are related to sporulation. Since the 1832 genes were selected based on >10-fold induction, our use here of a two-fold threshold likely underestimates the number of genes specific to sporulation.

**Fig 6:**
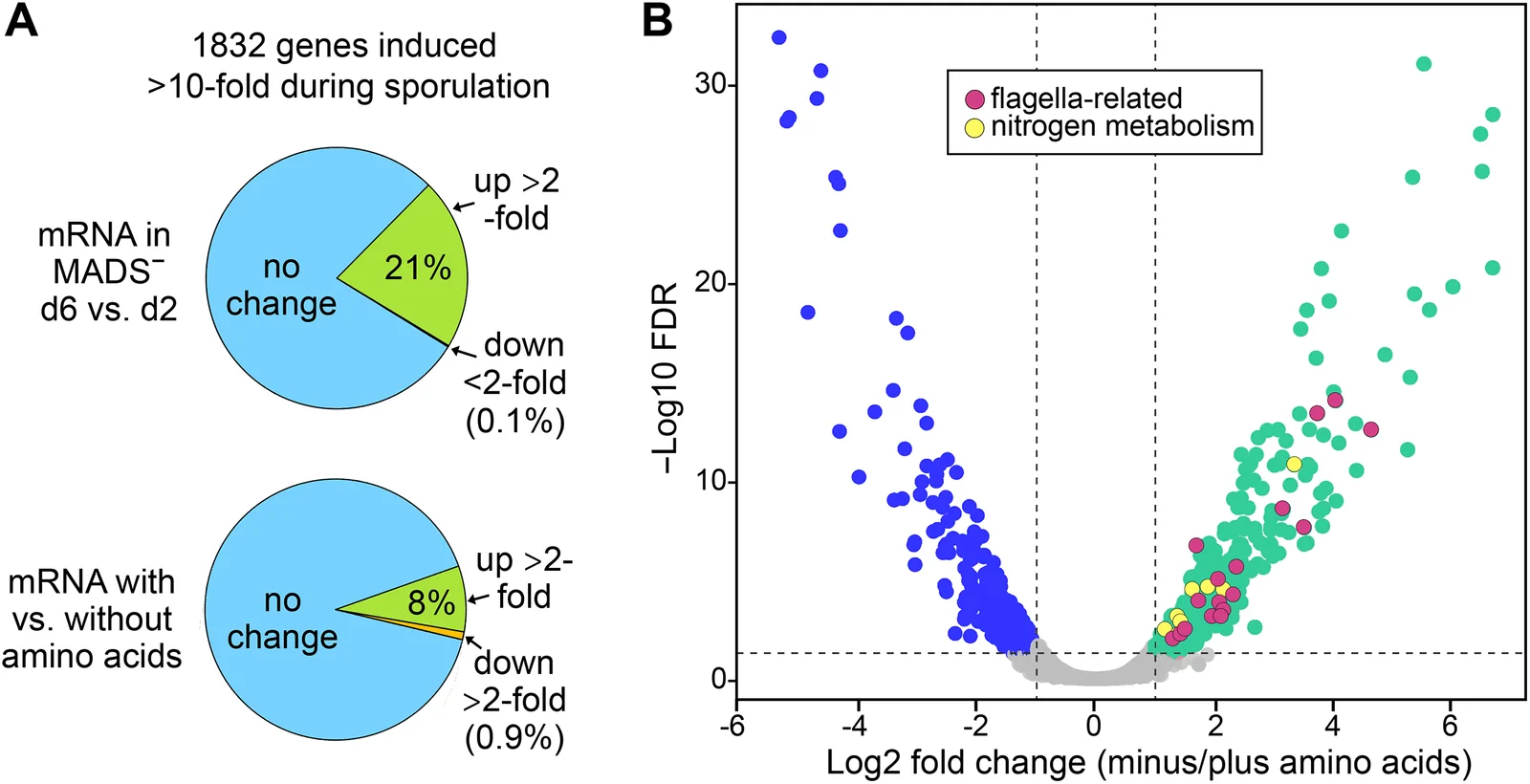
Most genes induced during *P. infestans* time courses are related to sporulation and not aging. **(A)** The top pie chart indicates that 79% of the 1832 genes up-regulated ^3^10-fold during sporulation were not up-regulated in 6-day versus 2-day rye-sucrose agar cultures of strains silenced for the MADS transcription factor. The bottom chart indicates that 92% of the genes are not up-regulated in synthetic media lacking amino acids compared to the same media with amino acids. All samples were grown in the dark. **(B)** Volcano plot of mRNA from the 1832 genes in synthetic media with or without amino acids. Yellow spots highlight genes involved in nitrogen metabolism that are expressed at higher levels in the absence of amino acids. Red spots mark 17 genes encoding flagellar proteins up-regulated by the absence of amino acids. Other flagellar genes (n = 200) that were not differentially expressed are not highlighted.

Our second approach relied on the hypothesis introduced above that limiting amino acids triggers sporulation. We therefore analyzed RNA-seq data from nonsporulating hyphae grown for 2.5 days in minimal media lacking amino acids (containing ammonium sulfate) or with 1% casamino acids (Fig 6A bottom and 6B). Only 8% of the 1832 genes were up-regulated by two-fold or more in the amino acid-lacking media and therefore might be more related to aging than sporulation. Since the 1832 genes were selected based on >10-fold induction, our use of a two-fold threshold likely overestimates the number of aging genes. Notable among the genes expressed more in media lacking amino acids were nitrate and nitrate reductase, the nitrate transporter, and ammonium transporters. These are portrayed by the yellow circles in Fig 6B. Such proteins are presumably needed since amino acids are the preferred nitrogen source for *P. infestans* and once depleted, metabolism turns to alternatives [48].

Based on these two analyses, we suggest that the fraction of the 1832 genes that are directly related to sporulation is probably closer to 92% than 79%. The 79% value is too low since MADS acts at an intermediate stage of sporulation [31]. The 92% might even be an underestimate since 17 of the genes expressed more in the media lacking amino acids encode flagella proteins (Fig 6B, red circles). If amino acid limitation triggers sporulation, these may be among the first to be induced.

### Most up-regulated mRNAs persist in *P. infestans* sporangia

Further insight into the up-regulated genes came from studying whether their mRNA levels were still elevated in sporangia. This was the case for about 80% of the 1832 highly-induced genes (S4 Fig). Many encoded components of the flagellar axoneme such as dyneins and microtubule motors, plus proteins needed to maintain energy homeostasis during swimming such as adenylate kinases, which regenerate ATP from ADP. This might seem to contradict studies that concluded that ungerminated sporangia contain all proteins needed for zoospore formation during germination [25]. However, persistence of these transcripts probably helps maintain the function and structural integrity of the flagella after germination. Their persistence may be attributed to either continued transcription in the sporangial nuclei or long half-lives.

In theory, sporulation-induced genes for which mRNAs did not persist may serve functions needed only early in the developmental pathway or may relate to aging. GO term analysis of the non-persistent mRNAs indicated enrichment for phosphatidylinositol binding, transporters, amino acid permeases, and nitrogen metabolism. The latter included the proteins for assimilating nitrate and ammonium mentioned earlier. Also among the non-persistent mRNAs were those encoding members of the NPP1 necrosis-inducing factor family, which have been associated with the necrotrophic stage of plant infection [49]. These are probably not required for sporulation *per se* but are developmentally programmed to be expressed late in the life cycle. Another non-persistent sporulation-induced mRNA encoded a Myb transcription factor (PITG_21177) that would not be needed in sporangia if its role was related to early spore development.

### Transcripts for many metabolic pathways decline during sporulation

Since nutrient limitation was hypothesized to trigger sporulation, we examined the metabolic transcriptome of *P. infestans* by examining KEGG pathways [50] and genes encoding each enzyme activity. The latter involved aggregating the FPKM values of genes assigned to the same KEGG orthology group, as more than half were members of families. In both rye-sucrose and tomato leaf experiments, most pathways showed reduced gene expression, which generally persisted in sporangia (S5 Fig). This is consistent with a modeling study that concluded that as infection progresses, *P. infestans* increasingly relies on host-derived nutrients rather than *de novo* synthesis [51]. Despite these declines, metabolic genes still accounted for ~12% of the transcriptome, as decreases in most genes were balanced by strong up-regulation of a few. The most prominent example was sugar alcohol dehydrogenases (*e.g.,* PITG_04121, KEGG identifier K00008), whose expression rose by more than 100-fold and constituted 12% of the metabolic transcriptome.

Attempts to place changes in metabolic gene expression in a biological context were complicated by disparity in how they were expressed in the rye-sucrose versus tomato time courses. Metabolic transcript levels in those experiments were only moderately correlated (*e.g.,* Pearson *r*^*2*^ = 0.70 and 0.71 for days 2 and 5, respectively) in contrast to non-metabolic genes (*r*^*2*^ = 0.93). This is not surprising since we had shown that metabolite levels during the rye-sucrose media and tomato experiments were often different (Fig 2).

Nevertheless, some consistent changes were observed in the rye-sucrose media and tomato time courses. Most enzymes displayed lower mRNA levels in sporangia compared to hyphae (S5 Fig). For example, reductions of about 50% were seen for most genes in the TCA cycle (*e.g.,* isocitrate dehydrogenase, malate dehydrogenase, and succinate dehydrogenase), oxidative phosphorylation (*e.g.,* ATP synthase, cytochrome C oxidase, ubiquinol-cytochrome c reductase), and amino acid biosynthesis. This suggests that sporangia have stored enough metabolites to reduce the need for *de novo* synthesis and have shifted resources towards proteins needed for infection, such as effectors. This is consistent with one study that found that most amino acids were abundant in sporangia, even though most declined in the older hyphae [40].

Up-regulated in both the rye-sucrose and tomato time courses were most enzymes involved in inositol phosphate metabolism. These included phosphatidylinositol 3-kinase, phosphatidylinositol 4-kinase, 1-phosphatidylinositol-4-phosphate 5-kinase, 1-phosphatidylinositol-3-phosphate 5-kinase, and others. Products of these enzymes typically participate in signaling, membrane identity, and trafficking and are likely to be related to the morphological changes that occur during sporulation or the germination.

### Cyclic light-regulated expression of sporulation genes in *P. infestans*

Many sporulation-associated genes exhibited cyclic, light-influenced expression patterns in Fig 4. To better assess this, we calculated the mean daily change in mRNA levels for the 1832 strongly up-regulated genes across three consecutive light/dark cycles in the rye-sucrose study. Of these, 282 were classified as light-neutral since their mRNA levels did not oscillate (Fig 7A). The remaining genes displayed modest to substantial (>4-fold) variation. Since a minority of genes were light-neutral, light probably regulates an early step in sporulation but is not the primary inducer.

**Fig 7:**
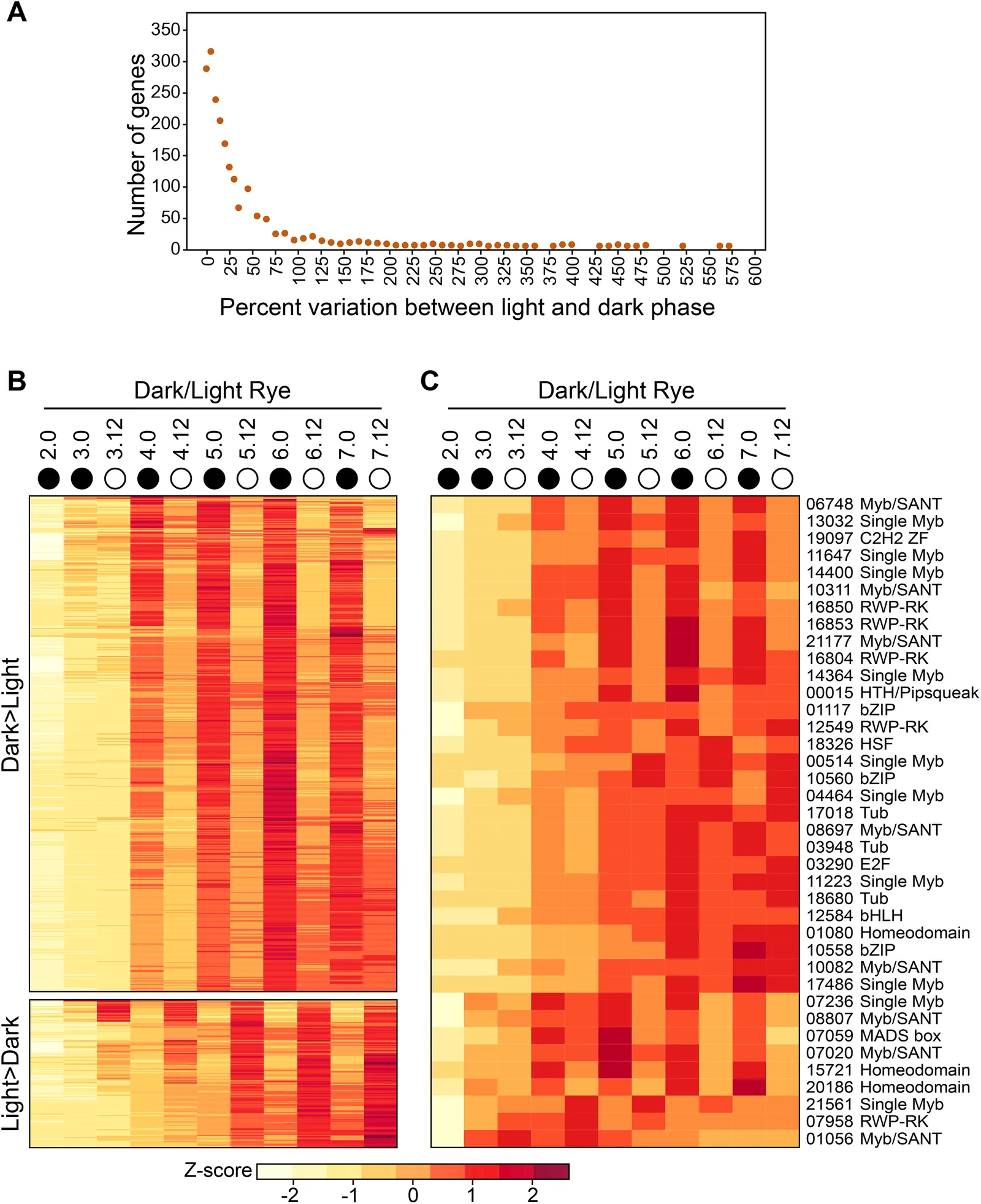
Many sporulation-induced genes in *P. infestans* exhibit cyclic light-influenced expression. **(A)** Cyclicity of the 1832 sporulation-induced genes averaged over three daily cycles. **(B)** mRNA levels of genes induced during sporulation that show consistently higher abundance in the dark (top panel) or light (bottom) phases. **(C)** mRNA levels of transcription factors induced ^3^4-fold during sporulation, labeled by their PITG number and family.

This pattern was examined in more detail by expanding the analysis to the 2936 genes induced by >4-fold in the time courses and focusing on those showing at least 15% variation over three light/dark cycles. Exhibiting higher transcript levels in the dark were 699 genes whereas 50 had higher mRNA levels in the light (Fig 7B). These numbers would rise if less stringent thresholds for cycle number or fold-change were applied. The functions of proteins encoded by the cyclic genes were diverse, similar to the overall set of sporulation-induced genes. However, a notable difference emerged when comparing the 282 light-neutral genes with the 282 genes showing the strongest cyclicity. Based on GO and KEGG term analyses, the light-neutral genes were highly enriched for flagella-related functions, whereas the most cyclic genes were enriched for metabolic enzymes. These findings support a model in which the regulator responding to nitrogen limitation acts upstream of the light-sensing regulator and activates expression of many of the flagella-associated genes.

Since transcription factors likely are responsible for the oscillating mRNA levels, we examined which were sporulation-induced and cyclic. Of the 197 genes predicted to encode transcription factors in *P. infestans* that were expressed above the count threshold [52], 39 were induced >4-fold during sporulation and nearly all fluctuated with light. Most had higher mRNA levels in the dark, although the timing of their expression varied (Fig 7B). For example, Myb PITG_01056 and RWP-RK PITG_07958 showed peak expression around day 3, while others peaked around day 5 (*e.g.*, MADS factor PITG_07059) or 7 (*e.g.*, Myb PITG_04464). Adding in genes with higher expression in the light (*e.g.*, heat shock factor PITG_18326), we can identify at least four stages of light-regulated regulation of the sporulation program.

We also checked the cyclic genes for transcription cofactors. This identified PITG_15041, which encodes a dark-induced histone methyltransferase that might act with cyclic or non-cyclically expressed transcription factors. We also identified 19 protein kinases, a protein phosphatase, and a protein phosphatase regulatory subunit which exhibited cyclic expression and might regulate transcription factors. However, some genes encoding sporulation-induced regulatory proteins did not display cyclic expression. This included Cdc14 protein phosphatase, which we have shown is required for sporulation [32].

### Humidity tightly regulates sporulation but loosely regulates transcription

In the experiments with *P. infestans* described above, culture plates or plants were kept in plastic bags or boxes with tight-fitting lids that preserved high relative humidity (RH). To study how humidity affects gene expression and sporulation, we constructed environmental chambers in which RH could be controlled. For the high-humidity chamber, dry medical-grade air was passed through a series of three water bubblers, producing 99–100% RH in the chamber. The low RH chamber was fed directly by the same dry air at the same flow rate. While the RH of the input air was < 1%, evaporation from the agar plates resulted in a stabilized RH of 34%, although RH at the hyphal surface was probably slightly higher due to boundary layer effects [53]. Lower RH could be achieved by increasing airflow, but this caused excessive drying of the cultures.

For our experiments, membranes inoculated with sporangia were laid upon rye-sucrose agar and kept at high RH for two days to permit germination and initial hyphal growth. Cultures were then divided between high and low RH chambers. Consistent with prior reports, sporangia formed only at high RH (Fig 8A). Cultures grown at low RH had a flat appearance, lacking the aerial hyphae and sporangiophores produced at high RH. However, when low-RH cultures were moved to high RH on day 5, fuzzy aerial growth soon became visible followed by sporangia. It follows that humidity regulates a developmental step preceding sporangiophore formation and that cultures maintained at low RH are primed to sporulate when a favorable RH occurs.

**Fig 8:**
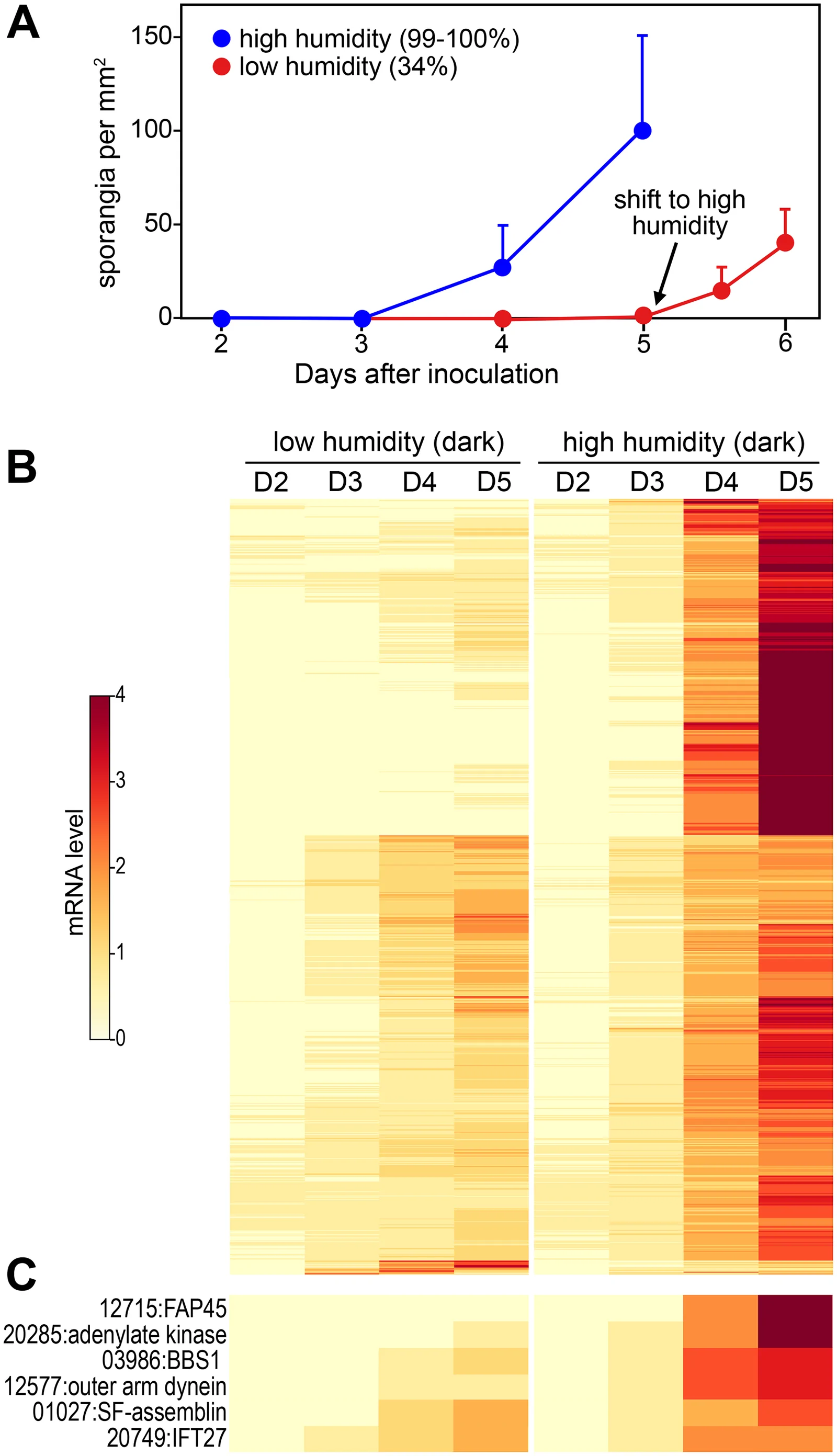
A majority of sporulation-induced *P. infestans* genes are partially up-regulated under low humidity. **(A)** Sporulation under high or low humidity. The data at days 5.5 and 6 are low-humidity cultures that were shifted to high humidity on day 5. **(B)** Expression of the 1832 sporulation-induced genes under the low and high humidity regimes in the day 2–5 timepoints shown in panel A. **(C)** Same but highlighting selected genes described in the text.

To interpret how humidity affected the transcriptome, we extracted RNA from the low and high RH time courses and examined gene expression using RNA-seq (S5 Table). Not surprisingly, all the 1832 strongly-induced genes from the earlier experiment were up-regulated during the high RH time course. However, at low RH three distinct patterns of expression emerged (Fig 8B). About 25% of genes showed similar expression under both humidity conditions, 20% were not significantly up-regulated under low RH, and the remainder displayed partial induction. Each class included genes directly related to sporangium development, as illustrated for six representative genes in Fig 8C. For example, induced only at high RH was the gene encoding the ortholog of FAP45, a protein that binds within the outer doublets of the axoneme. At the other extreme, the gene for intraflagellar transport protein 27 was induced similarly at high and low RH. An intermediate pattern was observed for the flagellar rootlet protein SF‑assemblin, which showed partial induction at low RH. Together, these results support the idea that humidity regulates a relatively late checkpoint in sporulation, and while required for activating some genes, high RH may also contribute to the continued expression of some genes induced earlier in the program.

While humidity presumably affects a transcription factor that controls sporulation, this likely occurs post-transcriptionally. Excluding those associated with sporulation, few *P. infestans* genes were up or down-regulated more than two-fold between low and high RH, including transcription factors. Among the few affected by RH were multiple genes encoding catalase-peroxidases (*e.g.,* PITG_05579), as their mRNA levels rose 5–10-fold at low RH. The other major type of gene affected by humidity were a family of nine (*e.g.,* PITG_13333) that consistently exhibited five-fold higher mRNA levels at low RH. Their predicted protein products were specific to Peronosporalean oomycetes but lacked clues to their function.

### A cryptochrome regulates sporulation in *P. infestans*

*P. infestans* encodes three cryptochromes with canonical photolyase and FAD-binding domains (Fig 9A). These are the only type of known light-sensing protein detected in *P. infestans* [15]. PITG_16100 and PITG_16104 contain an N-terminal extension absent in PITG_01718. This does not appear to be due to a truncated gene model since the transcription start site was confirmed by 5'MACE analysis.

**Fig 9:**
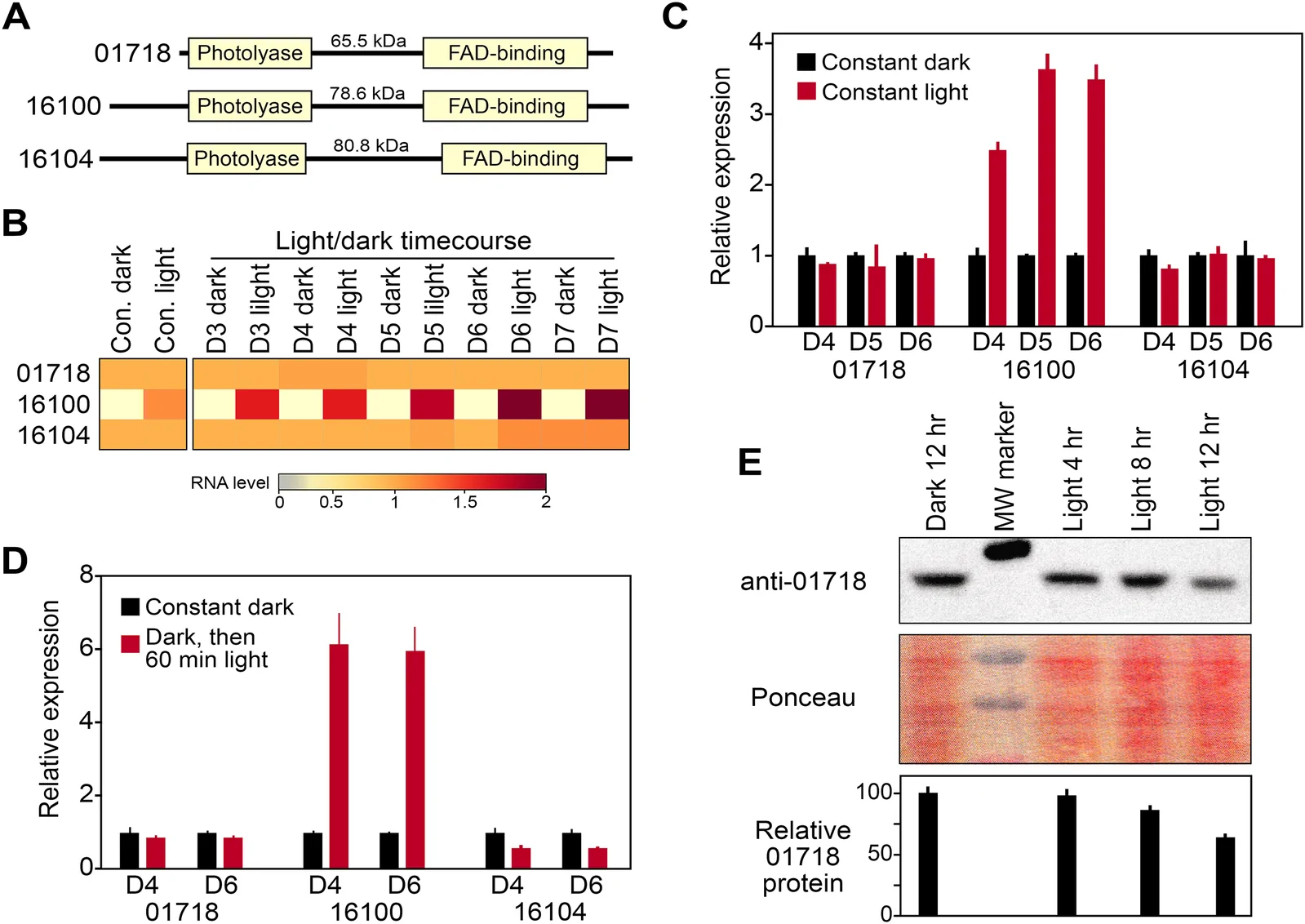
*P. infestans* encodes both light-induced and light-neutral cryptochromes. **(A)** structure of the three proteins including their functional domains. **(B)** RNA-seq expression patterns on rye-sucrose media after four days of constant darkness, constant white light, or after 12 h exposure to light or dark in a time course. Data are normalized to an average of 1.0 for each gene. **(C)** mRNA levels for the three genes in 4, 5, or 6-day cultures grown in darkness or under constant white light, as determined by RT-qPCR using tissue samples independent of those used for RNA-seq. Data are scaled to a value of 1.0 for the dark samples. **(D)** Effect of 60 minutes of white light on mRNA levels, based on RT-qPCR. **(E)** Effect of light on levels of PITG_01718 protein. Protein was extracted from hyphae grown in the dark for 12 hr, and from cultures exposed to white light for 4, 8, or 12 hr. The protein was detected using anti-01718 antibody and corrected for loading variation using Ponceau staining.

Based on RNA-seq, the mRNA levels of PITG_01718 and PITG_16100 were similar in cultures grown in constant light, constant darkness, and in both phases of a light/dark cycle (Fig 9B). In contrast, transcripts of PITG_16100 were consistently higher in light. These patterns were confirmed by RT-qPCR (Fig 9B). Since certain cryptochromes in other species are induced by short bursts of light, but level off expression in constant light due to acclimatization [54], we checked the effect of a 60 min pulse of light on cultures grown previously in the dark for four or six days (Fig 9D). PITG_16100 transcripts were induced six-fold after this short exposure compared to the constant dark samples, PITG_16104 expression was reduced by about 45%, and PITG_01718 transcripts were not significantly affected.

In addition to transcriptional control, some cryptochromes in other organisms are regulated at the level of protein stability [55]. To test this in *P. infestans,* we focused on PITG_01718 since this had exhibited light-neutral levels of mRNA. Using Western blot analysis, we found that the level of the protein declined when cultures were exposed to light in a time-dependent manner (Fig 9E).

Whether the cryptochromes influence sporulation was tested using gene silencing, by expressing hairpin RNAs in transformants. This can result in a transcriptional knockdown due to heterochromatinization of the targeted gene [56]. Knockdown transformants were obtained for each gene (Fig 10A). For PITG_01718 we obtained one strong (>99%) knockdown transformant and two with partial (60–75%) knockdowns. All exhibited reduced sporulation, roughly in proportion with the level of knockdown (Fig 10B). For example, strain Sil9 showed a 99% reduction of both cryptochrome expression and sporulation, while strain Sil17 had a 76% reduction in expression and made 94% fewer sporangia. The reduction of sporulation was observed regardless of whether the strain was grown in white, red, or blue light or in the dark, which may indicate that the effect of light on sporulation is mediated by one of the other cryptochromes (Fig 10C). The silenced strains retained the ability to colonize tomato leaves if hyphal plugs were used as the inoculum. Since we have observed that the heterochromatic state induced by a hairpin RNA sometimes spreads to neighboring genes [57], we tested the genes flanking PITG_01718 in the silenced strains (S6A Fig). Both were expressed normally, indicating that the lack of sporulation results from the knockdown of PITG_01718, which appears to be a positive regulator of sporulation.

**Fig 10:**
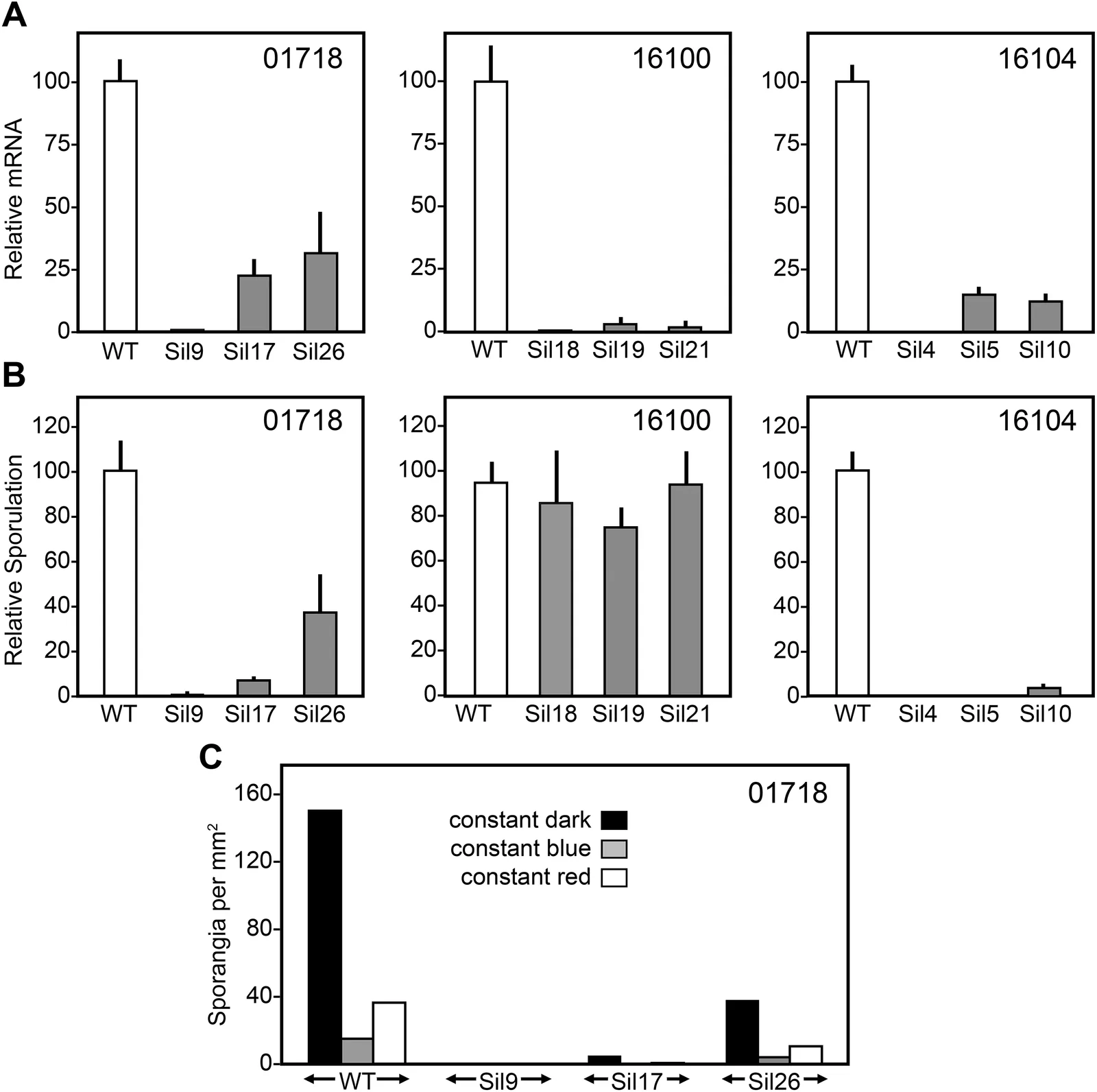
Silencing cryptochrome genes in *P. infestans* blocks sporulation. **(A)** mRNA levels in the progenitor strain of *P. infestans* (WT) and three representative transformants, determined using qRT-PCR. Expression levels are expressed as percent relative to WT. **(B)** Sporulation of each strain relative to wild type in cultures exposed to a 12 h white light/dark cycle. **(C)** Density of sporangia in wild type and three strains with knockdowns of PITG_01718 grown in darkness or under blue or red light.

For PITG_16100, we obtained several transformants that exhibited strong (>95%) silencing (Fig 10). Normal rates of sporulation were observed in both light and dark-grown cultures. The genes flanking PITG_16100 maintained normal expression (S6A Fig).

Silenced transformants were also obtained for PITG_16104, and all exhibited poor sporulation (Fig 10). While the left-flanking gene, PITG_16103, was expressed at normal levels in the knockdowns a gene to the right appeared to be co-silenced (Fig S6A). Consequently, either PITG_16104 or this other gene might be responsible for the sporulation defect. The right-flanking sequence had not been annotated as a gene in the reference genome, but matched reads in RNA-seq data (S6B Fig). The region could be translated into a 266 amino acid protein that has orthologs in other *Phytophthora* spp. and has a predicted N-terminal lipid anchor but no other known functional domains. Attempts to evade co-silencing by targeting hairpin RNAs to the 5’-most region of PITG_16104 were unsuccessful.

We also performed overexpression experiments by expressing the coding sequences behind the strong *ham34* promoter with a C-terminal FLAG tag. As shown in S7 Fig, over-expressing strains were obtained for all three genes. Their expression levels ranged from about 20–90 times higher than those of the native loci based on qRT-PCR, and production of the proteins was confirmed by anti-FLAG immunoblotting. However, overexpression produced no consistent effect on sporulation density in cultures grown under either light or darkness. We also repeated the experiment with a N-terminal *myc* tag. Strains expressing *myc*-tagged PITG_16104 were obtained and found to sporulate normally. Attempts to obtain viable strains expressing *myc*-tagged PITG_01718 or PITG_16104 were unsuccessful.

The primary light-sensing chromophore in cryptochromes is FAD, which after being reduced by light causes a conformational change that affects signaling [58]. Some cryptochromes also bind pterins, which harvest shorter wavelengths than FAD and transfer the captured energy to FAD. It was therefore interesting that metabolomic analysis indicated that FAD and pterin levels in the *P. infestans* hyphae fell by 66 and 64%, respectively, when comparing day 5 to day 2 in the sporulation time course. Whether these changes affect the cryptochromes is unknown.

### Sporulation-associated transcription is regulated divergently in *P. capsici* and *P. infestans*

To confirm prior reports [16] that light stimulates sporulation in *P. capsici* and establish conditions for further experimentation, sporulation was measured under different light regimes. This employed V8 Juice media since little sporulation occurred on the rye-sucrose agar used with *P. infestans.* As shown in Fig 11A, light was essential for sporulation in *P. capsici,* opposite to the case with *P. infestans.* Moreover, when cultures were treated with a light/dark cycle, sporulation was retarded in the dark phase. An additional difference between *P. infestans* and *P. capsici* was that only blue light stimulated sporulation in *P. capsici,* while red and blue affected *P. infestans* (Fig 11B).

**Fig 11:**
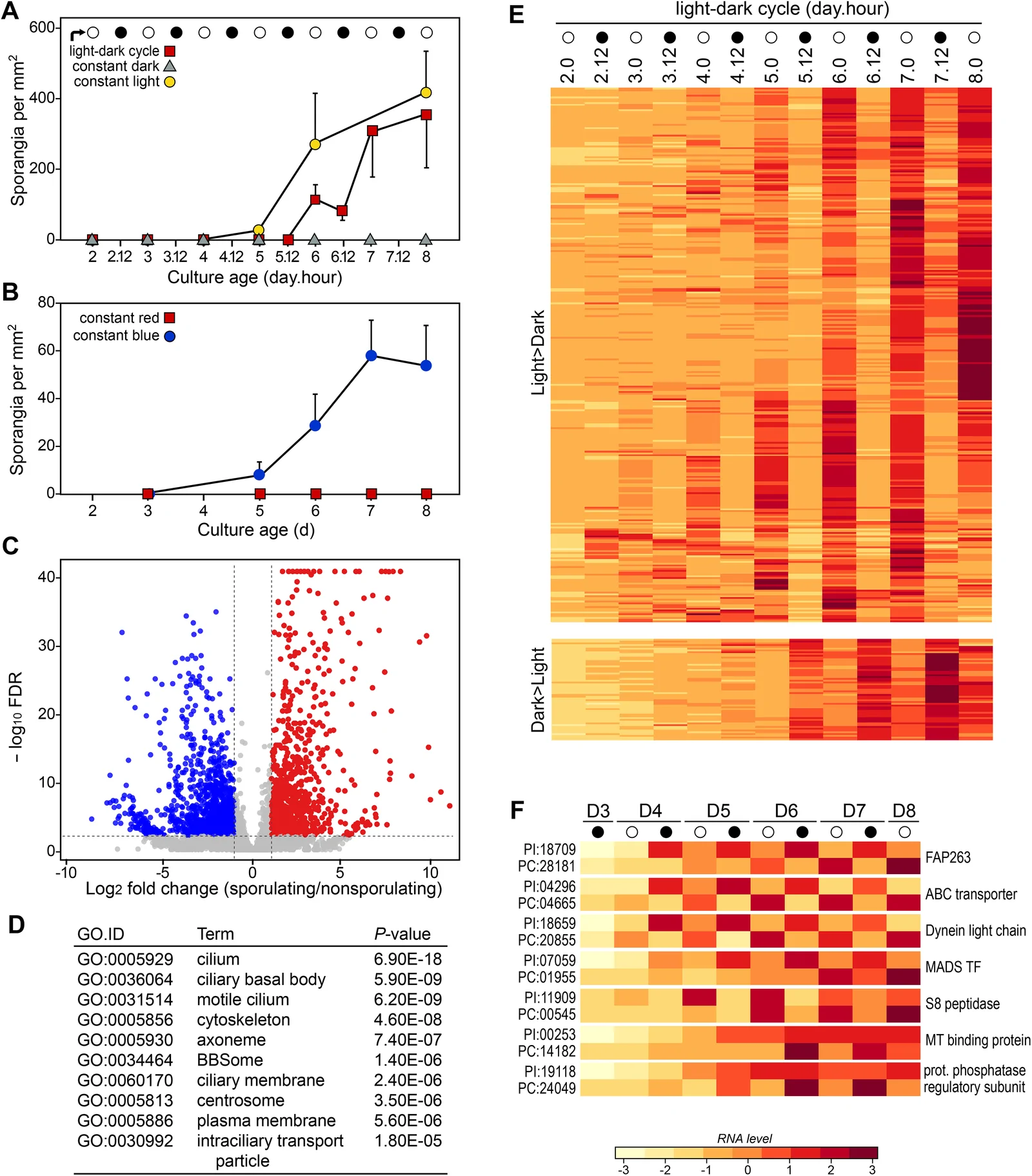
Many sporulation genes in *P. capsici* exhibit light-influenced expression patterns distinct from their *P. infestans* orthologs. **(A)** Sporangia density in cultures grown under a 12 h light/dark cycle, continuous white light, or continuous darkness. **(B)** Effect of red and blue light on sporulation*.* **(C)** Comparison of gene expression in 7-day versus 2-day cultures (sporulating and nonsporulating, respectively) grown with a light/dark cycle. **(D)** The top over-represented GO terms for genes up-regulated during sporulation. **(E)** Genes up-regulated during the light/dark time course that exhibit cyclic expression with higher levels in the light phase (top) or dark phase (bottom). The data are shown as a *z*-score heatmap, which represents the number of standard deviations a value is from its row mean. **(F)** Patterns of expression of selected *P. infestans* genes (PI) and their *P. capsici* (PC) orthologs during light/dark time courses.

Gene expression by *P. capsici* during the light/dark time course was measured by RNA-seq. As with *P. infestans,* many genes were differentially expressed (Fig 11C, S6 Table). Based on edgeR analysis 1822, 1016, and 488 genes were up-regulated by ^3^2, ^3^4, or ^3^10-fold using an FDR cutoff of 0.05, while 1130, 481, and 194 were down-regulated by those amounts. These are fewer than observed for *P. infestans,* but we believe this is since sporulation was less intense and more variable within the *P. capsici* cultures. Nevertheless, most GO terms that were over-represented among the sporulation-induced genes were related to flagella, as with *P. infestans* (Fig 11D). Also, a majority of *P. capsici* orthologs of the 1832 sporulation-induced *P. infestans* genes were up-regulated in the *P. capsici* time course.

Many *P. capsici* genes exhibited cyclic expression during the light/dark time course. However, while most cyclic genes in *P. infestans* showed higher transcript levels in the dark, the opposite was usually true in *P. capsici.* As shown in Fig 11E, 300 of the *P. capsici* genes induced in the time course by >4-fold had mRNA levels higher in the light while 57 were higher in the dark. As with *P. infestans*, these values will vary based on the thresholds applied for calculating cyclicity.

When comparing the cyclic nature of *P. capsici* and *P. infestans* genes*,* the most common observation was that their orthologs were induced in cultures of similar age relative to the onset of sporulation, but with light and dark phases reversed. Examples shown in Fig 11F include genes encoding the flagella protein FAP263, a dynein light chain, and an ABC transporter. Other genes displayed the same inverted light response but with different timing. For example, the MADS-box transcription factor was induced much earlier in *P. infestans* than *P. capsici.* A third pattern was that the cyclic expression sometimes occurred only in one species. This is illustrated in Fig 11F for genes encoding a microtubule binding protein and a phosphatase regulatory subunit. Together, these results indicate substantial rewiring of the mechanism that regulates sporulation-related transcription between the two species.

Other analyses indicated that while light tightly controls sporulation in *P. capsici,* it regulates only part of the underlying transcriptional program. This conclusion was based on RNA-seq analysis of cultures grown in constant light or darkness (Fig 12, S6 Table). Although no sporulation occurred in the dark, most genes encoding flagella proteins (68%) were up-regulated with similar kinetics in both light and dark conditions. About 13% were expressed at higher levels in the light. Interestingly, 4% showed higher expression in the dark which suggests the presence of a light-activated repressor. A few genes showed little induction during either time course, but these typically belonged to large families (such as dyneins) for which the incorrect ortholog may have been identified. We conclude that as in *P. infestans,* light regulates an intermediate phase of sporulation, with most flagellar genes activated prior to the light-regulated checkpoint.

**Fig 12:**
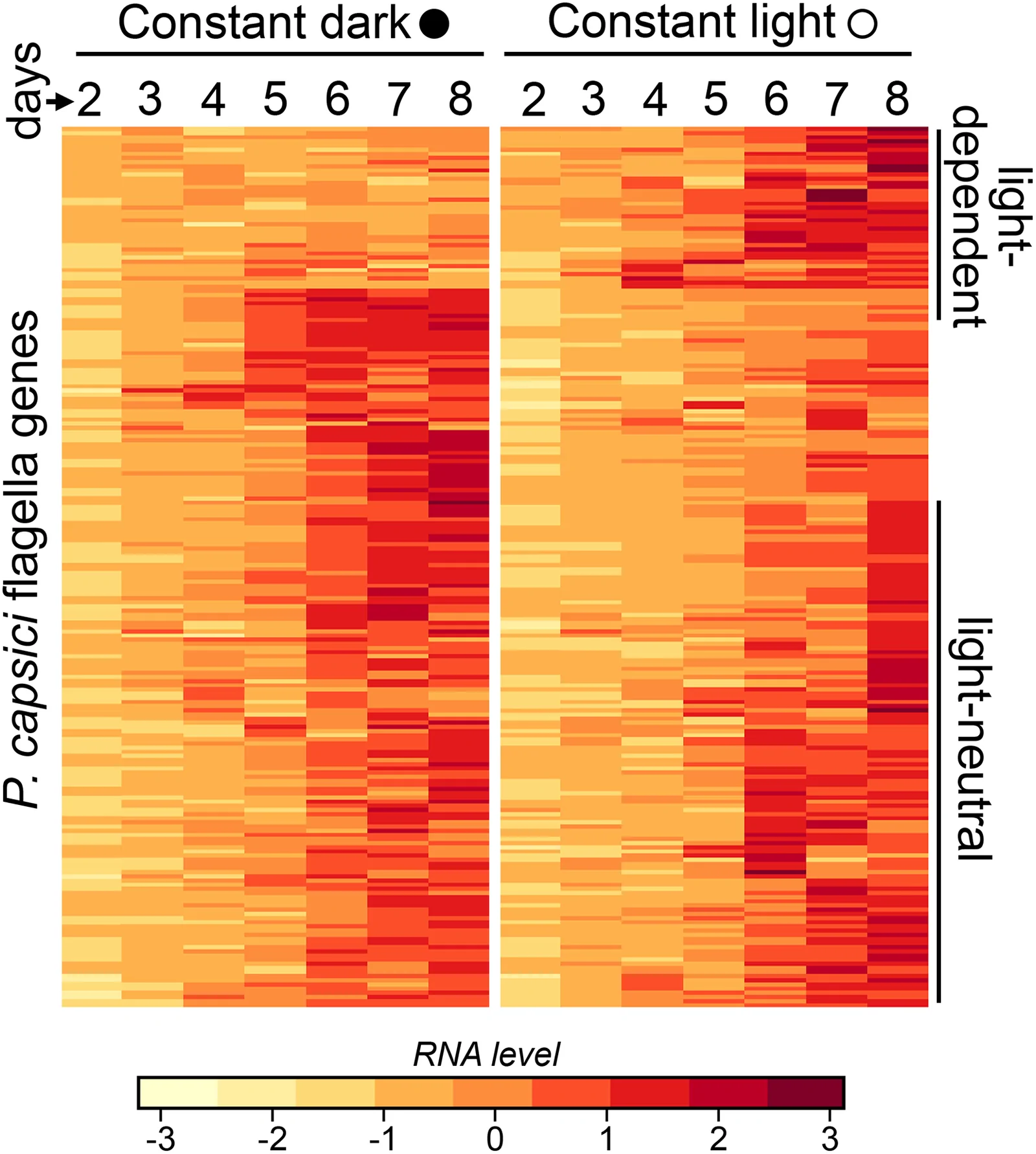
*P. capsici* genes encoding flagella proteins exhibit light-dependent or light-neutral patterns of expression. Expression of flagella-associated genes during growth in constant darkness (left) or light (right) over an eight-day time course. Indicated are 212 genes showing orthology with sporulation-induced *P. infestans* flagellar genes from a prior study [98]. RNA levels are shown as a *z*-score heatmap.

### *P. capsici* and *P. infestans* cryptochromes have diverse evolutionary trajectories

To better understand how light affects *P. capsici,* we searched its genome for genes encoding light-sensing proteins. While three cryptochromes were found in *P. infestans,* only two were detected in *P. capsici.* This difference was consistent between independent assemblies of both species. Similar to the *P. infestans* proteins except PITG_01718, the *P. capsici* proteins were larger than cryptochromes from plants and animals due to an extended N-terminal region (S8A, S8B Fig). As shown in S8C Fig, one *P. capsici* gene (DVH05v4_014408) was up-regulated by about six-fold by light while the other (DVH05v4_014404) was light-neutral.

To further explore the cryptochrome family, we conducted a phylogenetic analysis of oomycetes and other taxa. Cryptochromes from oomycetes in the order Peronosporales, which includes *Phytophthora,* formed a clade sister to animal-type cryptochromes (S8D Fig). In contrast, cryptochromes from *Aphanomyces,* a species within the order Saprolegniales, clustered within the animal clade. Other saprolegnians such as *Saprolegnia* spp. lacked cryptochromes, although like all oomycetes they contained members of the related photolyase family.

The Peronosporalean cryptochromes grouped into two strongly supported clades, which are labelled A and B in the supplemental figure. Intriguingly, the light-neutral gene from *P. capsic*i (DVH05v4_014404) and the light-induced gene from *P. infestans* (PITG_16100) were both in clade B. Also remarkable was that PITG_16100 and PITG_16104 are closely linked on the chromosome, inferring evolution from a common ancestor, yet have diverged in both sequence and regulation since only PITG_16100 is light-induced. The same is true for the *P. capsici* genes, DVH05v4_014404 and DVH05v4_014404.

*P. infestans* was unusual among oomycetes in encoding three cryptochromes, as most other species produced only two. One exception was *Phytophthora ramorum,* which possessed two proteins in clade A and one in clade B; these numbers were consistent between independent assemblies. Another exception involved the downy mildews *Bremia lactucae* and *Peronospora effusa*, which lacked clade A cryptochromes but had two clade B proteins. Like *P. infestans,* light suppresses sporulation in these species [59,60]. We conclude that is no direct relationship between the number or phylogenetic distribution of cryptochrome genes and whether light stimulates or represses sporulation.

## Discussion

Based on our studies of gene expression under different nutrient, light, and humidity regimes we can propose how these signals regulate sporulation in *P. infestans:* the process is triggered by a sensor of nutrient or metabolite levels, followed by a light-dependent checkpoint involving a cryptochrome, and then by a checkpoint that monitors humidity. It is unlikely that these signals act in a simple linear pathway to control sporulation since our data instead point to a branched network. For example, humidity regulated only a subset of sporulation-associated genes. Our comparative studies with *P. capsici* point to significant remodeling of the program between species, due at least in part to their different responses to light. Among the several thousand genes induced consistently in the artificial media and *in planta* time courses with *P. infestans* were multiple transcription factors, protein kinases, and other proteins that may contribute to the regulation of the transcriptional program, although at present we can not distinguish between genes needed to establish the sporangia and those expressed to prepare for germination. Prior studies have indicated that all proteins needed for zoospore release and encystment are already formed when sporangia have matured [24,25].

It is not surprising that declining nutrient levels appear to initiate sporulation, since in fungi carbon starvation is a common inducer of sporulation [8,61]. However, our data point to low concentrations of amino acids and not carbon sources as drivers of sporulation in *Phytophthora*. Most amino acids fell rapidly during our time courses in artificial media, but in tomato this occurred only for asparagine and to a lesser extent lysine. Combined with our observation that exogenous asparagine was the most effective amino acid at delaying sporulation, it is reasonable to hypothesize that the nutrient sensor has evolved to detect this amino acid. However, the signal from asparagine may be integrated with signals from other compounds. Asparagine may not be the sole regulator since it can be converted into other amino acids, and several other amino acids were observed to delay sporulation in our media manipulation experiments. A role of lysine is possible but uncertain since it appeared toxic in our experiment.

Regardless of which amino acid(s) trigger sporulation, this may be sensed by binding an enzyme, transporter, G protein-coupled receptor, or other regulatory protein such as a transcription factor. This could also involve a single protein capable of perceiving multiple ligands, as there are precedents in other taxa of proteins able to sense several amino acids [42,62,63]. The effect of amino acid limitation may also be indirect, for example through ribosome stalling caused by a deficiency in a charged tRNA [64].

We assume that light regulates an intermediate step in sporulation, since not all sporulation-induced genes exhibited cyclic behavior in the light/dark treatments. Whether light stimulates or represses sporulation in *P. infestans* and *P. capsici* can be related to their pathogenic lifestyles. *P. capsici* is primarily a soilborne pathogen that causes root, crown, and fruit rots, although it can occasionally form lesions on leaves [65]. Its sporangia are dispersed mostly by flowing water or rain splashes [66]. Its requirement for light to sporulate is logical since sporangia formed below ground would have limited opportunities for dissemination to new hosts. However, hyphae that reach the crown from an infected root would encounter light, triggering sporangium production above ground where their dispersal is more likely. In contrast, *P. infestans* is primarily a wind-transmitted foliar pathogen in which light restricts sporulation to the night. This is advantageous since fluctuations in temperature, humidity, and air flow after dawn promote sporangium detachment, and since water films conducive to zoosporogenesis are most likely to form when humidity peaks before dawn [22,67]. Nocturnal sporulation also benefits *P. infestans* since oomycete sporangia lack pigments for blocking ultraviolet light and are prone to desiccation during the day [26]. These constraints are less severe for *P. capsici* since its sporangia mostly travel in rain or irrigation water that provide protection [66].

The connection between light response and lifestyle appears to extend across *Phytophthora*, based on a survey of the literature. As shown in Fig 13, there is a perfect correlation between whether light stimulates or represses sporulation and whether a species is principally a pathogen of roots or leaves, although light has been examined systematically in only a limited number of species [16,68–72]. Light also suppresses sporulation in downy mildews, which are oomycetes that mostly infect foliage and are thought to have evolved from a *Phytophthora* ancestor [60,73,74].

**Fig 13:**
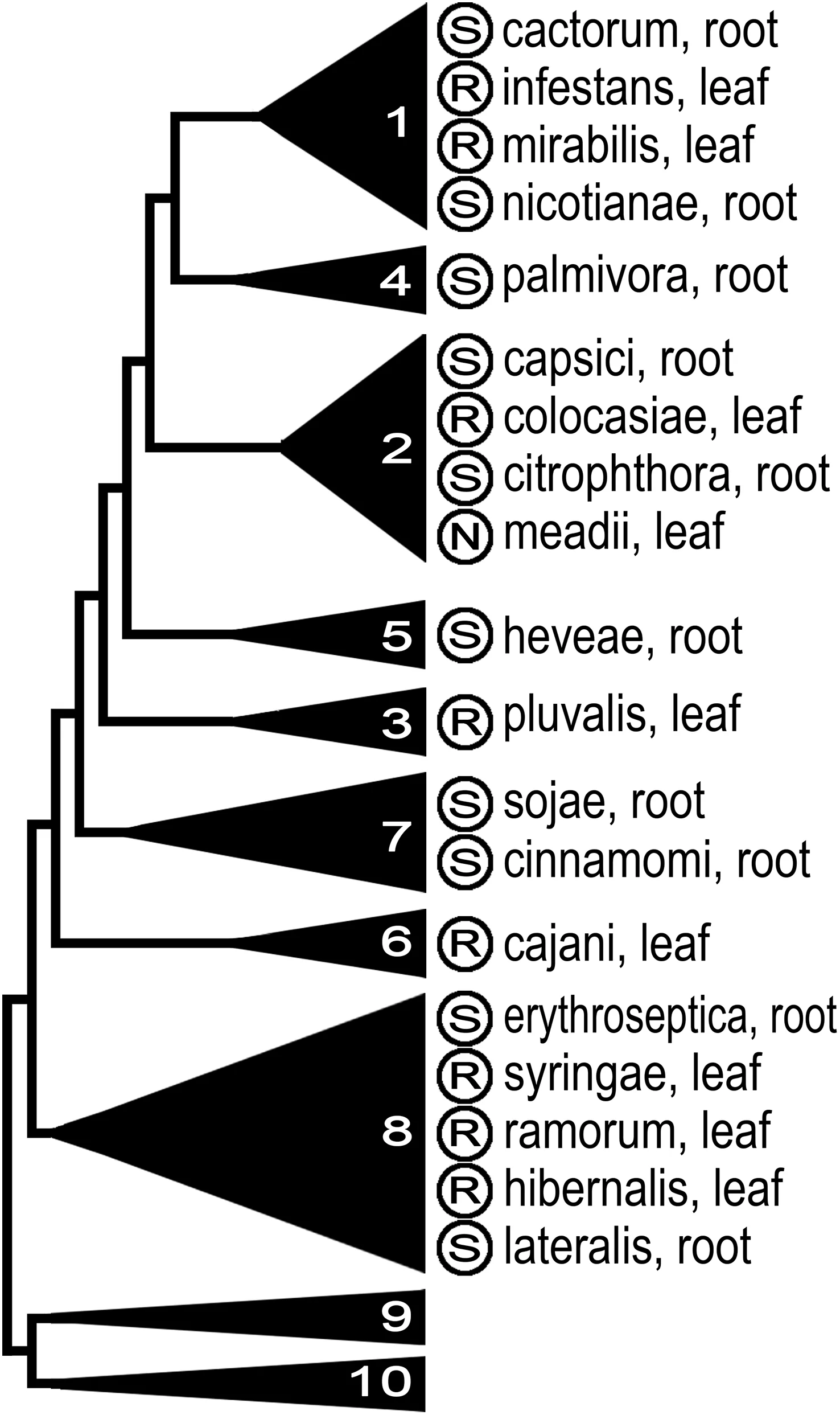
The effect of light on sporulation in *Phytophthora* is correlated with pathogenic lifestyle. Each species is labelled with S, R, or N indicating if light stimulates, represses, or has little effect on sporulation, respectively. The labels also indicate if the species are mainly root/crown pathogens or leaf/stem pests. The species tree is adapted from a prior multilocus analysis [99]. The phenotype for *P. cajani* is tentative since only a single light/dark cycle was tested [71]. No data are available for clades 9 and 10, although one study indicated that leaf pathogens in clade 10 sporulate in the dark [70].

Interestingly, how light affects transcription in *P. infestans* versus *P. capsici* is not as simple as transforming light-induced genes in one species into light-suppressed genes in the other. Although this pattern was common, some orthologs maintained the same responsiveness to light or were expressed with divergent timing. Such differences are not surprising since spore development in the two species is not identical. For example, *P. capsici* makes an unbranched sporangiophore bearing a single sporangium, while the sporangiophores of *P. infestans* are longer with multiple branches. *P. infestans* sporangia are also more elongated, form shorter pedicels, and typically release fewer zoospores than those of *P. capsici.* Even though the species belong to sister clades in molecular phylogenies [75], only about 60–70% of their genes could be assigned to shared ortholog clusters, indicating ample evolutionary divergence.

Our studies of the *P. infestans* cryptochromes provide a mechanistic connection between sporulation and light. Why *P. capsici* responds only to blue light and *P. infestans* to both red and blue can be rationalized based on their niches in the environment. Light near the top of a plant canopy is mainly red with a small amount of blue, while blue predominates underneath the canopy [76]. As a foliar pathogen *P. infestans* would therefore experience more red light, while *P. capsici* would mostly be beneath the canopy where adaption to blue would be advantageous. Our results contradict an earlier report that *P. infestans* is insensitive to red light [14], but they are consistent with studies of downy mildews which also reported that both blue and red influence sporulation [60,73]. Nevertheless, explaining how a cryptochrome could be sensitive to both wavelengths is challenging even though such behavior was described in *Chlamydomonas* [77]. While the FAD cofactor of cryptochrome is mostly blue-sensitive, red light may affect its oxidation status and thereby affect activity [78]. We cannot rule out the possibility that the red light response observed in oomycetes is mediated by a photoreceptor other than a cryptochrome.

Extending our understanding of how the cryptochromes function in *Phytophthora* will require identification of their protein interactors. In plants and animals, cryptochromes regulate gene expression by binding transcription factors or inhibiting ubiquitin ligases that would otherwise degrade those factors [79]. Cryptochromes also interact with proteins that establish circadian rhythms, although we have not detected evidence for such behavior in *Phytophthora*. In filamentous fungi, cryptochromes act as negative regulators of sporulation, which is the opposite of their role in *P. infestans* [80,81]. This difference is not surprising given the evolutionary distance between oomycetes and taxa in which cryptochromes have been studied [10]. While cryptochromes occur in most eukaryotic taxa, the proteins have experienced substantial diversification throughout evolution [82].

While we have made progress in understanding how light influences sporulation, more work is needed to understand the effects of humidity. This is usually sensed in biological systems through its effect on membranes, either by modulating mechanosensitive ion channels [83] or, under low humidity, by increasing reactive oxygen species by disrupting electron transport [84]. The latter mechanism may relate to our observation that catalase genes were over-represented among the genes up-regulated in our low humidity experiment (FDR = 10^-3^). The effects of light and humidity are intertwined since relative humidity rises as temperatures fall at night, typically reaching the high levels needed for sporulation only many hours after sundown. This is consistent with a model in which sporulation by *P. infestans* may initiate at any time due to nutrient limitation, partially pauses until nightfall, and then culminate a few hours before dawn.

Although our experiments only tested the impact of humidity on *P. infestans* growing on rye-sucrose media, others have shown that the requirement for high humidity also holds for plant lesions [11]. Whether *P. infestans* within a leaf can detect low humidity in the surrounding air has been controversial since many botanists assumed that the substomatal space is normally at or near 100% relative humidity [85]. However, recent studies of several species demonstrated that gas exchanges between the substomatal space and the external atmosphere are sufficient to reduce internal humidity below the threshold required for sporulation on days with moderate ambient humidity [86,87]. Since the sporangiophores emerge from leaves through stomata instead of rupturing the cuticle, *P. infestans* can sample the external air before committing to complete spore development. This capability is further enhanced by the ability of *P. infestans* to manipulate guard cells so that stomata stay open starting a day before sporulation, even though the stomata would normally close at night [88,89]. Together with the influence of light on the timing of sporulation, these adaptations likely contribute to the success of *P. infestans* and relatives as pathogens.

## Materials and methods

### Strains and growth conditions

*P. infestans* strain 1306 was maintained at 18°C in the dark on rye-sucrose agar [90]. *P. capsici* LT1534 [91] was maintained on 10% V8 juice agar at 18–23°C in the dark. For most experiments, cultures were established by spreading 200 μl of a suspension of 2 × 10^5^ sporangia per ml in water on an 85 mm polycarbonate membrane (Sterlitech, 0.4 µm pore) laid on the surface of the media. The exception was the azaserine experiment, where cultures were inoculated with a plug of hyphae. Experiments were performed with a minimum of three biological replicates unless indicated otherwise. Sporangia counts were made by rubbing sporangia off the membrane with water and a glass rod, followed by analysis with a hemocytometer.

Light treatments were conducted in a Percival I-36LLVL chamber at 18°C, illuminated with cool white fluorescent lights (32–75 μmol m^-2^ sec^-1^, Sylvania F20T12 CW). Some experiments used blue and red LED bulbs at 100 μmol m^-2^ sec^-1^ (Energetic YGA03A44). The intensity and emission spectra of the light sources were measured with an Apogee PS-200 spectroradiometer (Apogee Instruments, Logan, UT). For RNA-seq studies*,* cultures were placed in a clear polystyrene box. Dark treatments were performed in the same chamber except that the cultures were placed in light-tight boxes or bags. Plant infection assays were performed in the same chamber using detached leaflets of cultivar New Yorker. These were inoculated by dipping in a suspension of 2 × 10^5^ sporangia/ml, placed on the surface of 0.8% water agar, and sealed in bags.

For controlled humidity studies, cultures were incubated in gasketed 1.2 cubic foot cabinets (Bel-Art Secador) modified to have intake and outflow gas ports. Compressed medical-grade air was split into two paths which were passed through each chamber with or without first passing through three sequential 400-ml bubblers (McKesson). Air flow for each chamber was maintained at 3.5 liters per minute. Humidity was monitored using traceable meters distributed throughout each chamber.

### Metabolomics

Cultures were initiated on top of polycarbonate membranes with sporangia as described above, except that the underlying agar media had been clarified by centrifugation. Four replicates were prepared for each timepoint. Hyphae were peeled off the membrane and frozen in liquid nitrogen, while the underlying agar was cut into small pieces before freezing. The samples were lyophilized and provided to Metabolon, Inc. for analysis (Durham, NC). Samples were processed by four ultra high-performance liquid chromatography/tandem accurate mass spectrometry (UHPLC/MS/MS) methods, and compounds identified by comparison with a library of metabolites using a proprietary pipeline. A similar approach was used with tomato leaves. Concentrations of each metabolite are reported in relative terms, *i.e.,* normalized by division by the sum of all detected metabolite signals.

Absolute amino acid concentrations in unused rye-sucrose media were determined from four samples of rye-sucrose media made over the course of a year, using different batches of grain. Samples were provided to the University of California-Davis Analytical Laboratory for protein removal followed by analysis on a Hitachi 8800 amino acid analyzer.

### RNA analysis

Tissue was harvested from cultures or leaves, ground to a powder under liquid nitrogen, and RNA extracted with the Plant Spectrum Total RNA kit (Sigma-Aldrich). Tissues from dark treatments were harvested in a dimly-lit room at approximately 0.03 μmol m^-2^ sec^-1^. For RT-qPCR, RNA was treated with RQ1 DNAse (Promega), cDNA made using the Maxima First-Strand cDNA Synthesis kit (Thermo-Fisher), and analyzed using the Dynamo HS SybrGreen kit (Thermo-Fisher) using a Bio-Rad CFX thermal cycler with the primers in S7 Table. Assays included reverse transcriptase-minus and template-minus controls and were performed with three technical replicates per biological replicate. Fold-change was calculated using the gene encoding ribosomal protein S3A for normalization, employing the delta-delta-Cq method and considering experimentally determined primer efficiencies.

For RNA-seq, three biological replicates were used except as indicated in Results. After checking for quality with a Bioanalyzer 2100 (Agilent Technologies), libraries for Illumina sequencing were prepared with the TruSeq strand-specific kit method (Illumina). Depending on the experiment, these were sequenced on an Illumina HiSeq4000 at University of California-Davis Genome Center to obtain 100-nt single-end reads or 75-nt single-end reads on an Illumina NextSeq500 at Cofactor Genomics (St. Louis, MO). For the humidity studies of *P. infestans,* each sample yielded an average of 105 million reads while for the light studies on rye-sucrose media this was 25.6 million reads. For plant infections, sequencing depth was based on a sliding scale which ranged from 143 million total reads for day 2–27 million reads for day 6. For studies of *P. capsici,* an average of 29.5 million reads were obtained. Reads were analyzed through the SystemPipeR pipeline [92], which included mapping the reads either to the *P. infestans* T30-4 or the *P. capsici* LT1534–2 reference genomes (Genbank accession GCA_000142945.1 and GCA_016618375.1, respectively; [93]). Differential expression was called using edgeR with TMM normalization, with a false discovery rate (FDR) threshold of 0.05 based on Benjamini-Hochberg statistics. Some analyses were repeated using edgeR or DESeq2 in Galaxy. Heatmaps were developed using heatmap2.

### Additional bioinformatics

To identify photoreceptors from oomycetes, a combination of BLASTP and domain-level searches were performed against sequences at fungidb.org. Gene models from *P. capsici* and *P. infestans* were corrected by examining RNA-seq data and, for *P. infestans,* using 5’ MACE [94] to confirm the start codons. Such analyses confirmed the start sites of the genes but falsified the introns called for PITG_16104 in the T30-4 genome. The RNA data also confirmed the gene models in our revision of the *P. capsici* LT1534 assembly. Protein domains were identified using the Conserved Domain Database of the National Center for Biotechnology Information (NCBI). Additional cryptochromes were obtained from the JGI Genome Portal and NCBI. Sequences were aligned using MUSCLE as implemented in Seaview, trimmed with the Transitive Consistency Score (TCS) program in the T-Coffee suite, and a Maximum Likelihood phylogeny calculated using PhyML with branch support measured by the approximate likelihood ratio test. To compare differentially expressed genes in *P. infestans* and *P. capsici,* orthologs were identified using Orthofinder, supplemented in some cases by reciprocal best Blast. GO term analysis was conducted with gProfiler [95] and GOHyperGAll within SystemPipeR [92].

### Gene manipulations in *P. infestans*

For knockdown experiments, 500-nt hairpins amplified from genomic DNA were cloned behind the *ham34* promoter in pSTORA, which places sense and antisense sequences upstream and downstream of the Ste20 intron [96]. For overexpression studies, constructs were made that added an N-terminal *myc*-tag (EQKLISEEDL) or a C-terminal FLAG-tag (DYKDDDDK), which were expressed behind the *ham34* promoter in pTOR. After confirming the constructs by sequencing, DNA for transformation was isolated using the Nucleobond Maxi kit (Machery-Nagel) and introduced into *P. infestans* by protoplast transformation [96]. Transformants were screened for silencing by RT-qPCR or expression by Western blotting using anti-*myc* or anti-FLAG.

### Immunoanalysis

Antibodies against PITG_01718 were raised in rabbit against the peptide SQTSKKRQREGSDDESKQQ. Antibodies against the FLAG and *myc* tags were purchased from Sigma-Aldrich and ThermoFisher, respectively. Proteins for blotting were extracted from five to seven-day-old hyphae by grinding under liquid nitrogen followed by extraction in 50 mM Tris pH 7.4, 30 mM NaCl, 1% sodium dodecyl sulfate (SDS), 1 mM dithiothreitol, 1 mM phenylmethylsulfonyl fluoride, and 20% glycerol. This involved (at room temperature) vortexing the tissue powder in the buffer for 30–60 sec followed by gentle rotation for 10 min at 60 rpm, and removal of debris by centrifugation for 10 min at 12,000 × *g*. Proteins were separated by SDS-10% polyacrylamide gel electrophoresis, electroblotted to nitrocellulose, and stained in 0.5% w/v Ponceau S containing 1% glacial acetic acid. After washing the membranes in phosphate-buffered saline containing Tween 20 to remove the stain, the membranes were incubated with the appropriate primary antibodies, incubated with peroxidase-labelled second antibody, and developed using the ECL detection kit (Cytiva) with a C-Digit scanner (Li-Cor).

## Supporting information

S1 Table Metabolomics data. (XLSX)

S2 Table Amino acid composition of rye-sucrose media. (XLSX)

S3 Table Expression levels (FPKM) of *P. infestans* genes in the time courses shown in Fig 4. (XLSX)

S4 Table Gene lists developed from the time courses in Fig 4 and used elsewhere in this study. (XLSX)

S5 Table Expression levels (FPKM) of *P. infestans* genes in the humidity study. (XLSX)

S6 Table Expression levels (FPKM) of *P. capsici* genes in the dark, light, and cyclic light time courses. (XLSX)

S7 Table Oligonucleotides used for cloning and PCR. (XLSX)

S1 Raw Images Raw images of gels and blots in the paper. (TIF)

S1 Fig Levels of carbon sources and metabolic intermediates in *P. infestans* hyphae and rye-sucrose media during the sporulation time course shown in Fig 2. (A) Sucrose, glucose, and glycolytic intermediates in media at days 0, 2, and 5 and in hyphae at days 2 and 5. (B) TCA cycle metabolites. The values are relative and should not be used to compare different compounds.
(PDF)

S2 Fig Assay of *P. infestans* hyphal extracts for asparaginase-glutaminase activity. One unit is defined as the amount of enzyme that liberates 1 µmole of ammonia per minute at 21°C at pH 7.5.
(PDF)

S3 Fig Density of *P. infestans* sporangia in cultures grown in rye-sucrose media under darkness, constant light, a light/dark cycle, or in tomato leaflets grown in a light/dark sample. The values come from cultures or tissues grown in parallel to those used for RNA-seq and shown in Fig 4. The inhibitory effect of light was less than that shown in Fig 3 since its intensity was lower than what was used in the prior figure.
(PDF)

S4 Fig Differential persistence of *P. infestans* mRNA in sporangia and sporulating hyphae. Shown are data from RNA-seq of sporangia purified from six-day cultures and hyphae (and sporangia when present) from days two to seven after inoculation. Data are normalized such that each row has a mean of 1.0. All tissues were grown in the dark.
(PDF)

S5 Fig mRNA levels of metabolic genes in time courses on rye-sucrose media (days 2–7 after inoculation) and tomato time courses (days 2–6), and sporangia (SP) harvested from those experiments on day 6. mRNA levels represented by the sum of FPKM values for each gene in the same KEGG orthology group, which are represented by the K numbers to the right of each heatmap. Data are normalized such that each row has a mean of 1.0.
(PDF)

S6 Fig Status of genes flanking the *P. infestans* cryptochromes in the knockdown transformants. (A) mRNA levels of genes flanking the three targeted loci, with expression determined by RT-qPCR and graphed relative to wild-type. PITG_01717 and PITG_01719 are left and right of PITG_01718, respectively, while PITG_16099 and PITG_16101 flank PITG_16100. With PITG_16104, the right flanking locus was not previously annotated and is labelled “new gene.” (B) RNA-seq reads aligned to the region of the chromosome containing PITG_16104, pointing out the previously unannotated gene on its right. The three strains shown in Panel A failed to sporulate and exhibited silencing of both the cryptochrome and the new gene. Hence, the sporulation defect might be due to the silencing of either locus. The PITG_16104-silenced strains also showed poor growth, which could also be due to either locus. A subset of this data was reported previously.
(PDF)

S7 Fig Effect of overexpression of *P. infestans* cryptochromes on sporulation. (A) Fold-change increase in total cryptochrome mRNA levels in transformants relative to the wild-type progenitor based on RT-qPCR. Each protein (PITG_01718, 16100, and 16104) was expressed behind the strong *ham34* promoter with a FLAG tag. (B) Sporulation in transformants after six days growth under light or dark conditions. Values are expressed as percent relative to the wild-type progenitor strain 1306 and are the mean of three biological replicates from each of two experiments performed in different weeks.
(PDF)

S8 Fig Cryptochromes in *P. capsici* and other oomycetes. (A) structure of the two cryptochromes from *P. capsici.* (B) Cryptochromes from human, *Arabidopsis thaliana,* and *Drosophila melanogaster.* Comparison with panel A shows that the oomycete proteins lack the N-terminal extension found in the other species. (C) Expression of the *P. capsici* cryptochromes in cultures exposed to constant light or darkness, four to six days after inoculation. (D) Phylogenetic tree showing cryptochromes from oomycetes, animals, plants, and the related DASH proteins. The *P. capsici* and *P. infestans* proteins are marked with arrows. A more limited analysis of cryptochromes drew similar conclusions about how the oomycete proteins clustered [15]. While standard cryptochromes have been shown to regulate plant and animal development, and circadian clocks, CRY-DASH proteins repair UV-damaged single-stranded DNA. Both types of proteins are blue-light receptors.
(PDF)
