# Supplementary material for: Transcriptional programs controlling sporulation in Phytophthora respond to nutrition, humidity, and cryptochrome-mediated light signals
Source: PLoS Pathog. 2026 Oct 12;22(10):e1014705. doi: 10.1371/journal.ppat.1014705 (PMC13674864; doi:10.1371/journal.ppat.1014705)
Supplement: Supplementary file 1 [file ppat.1014705.s009.pdf]

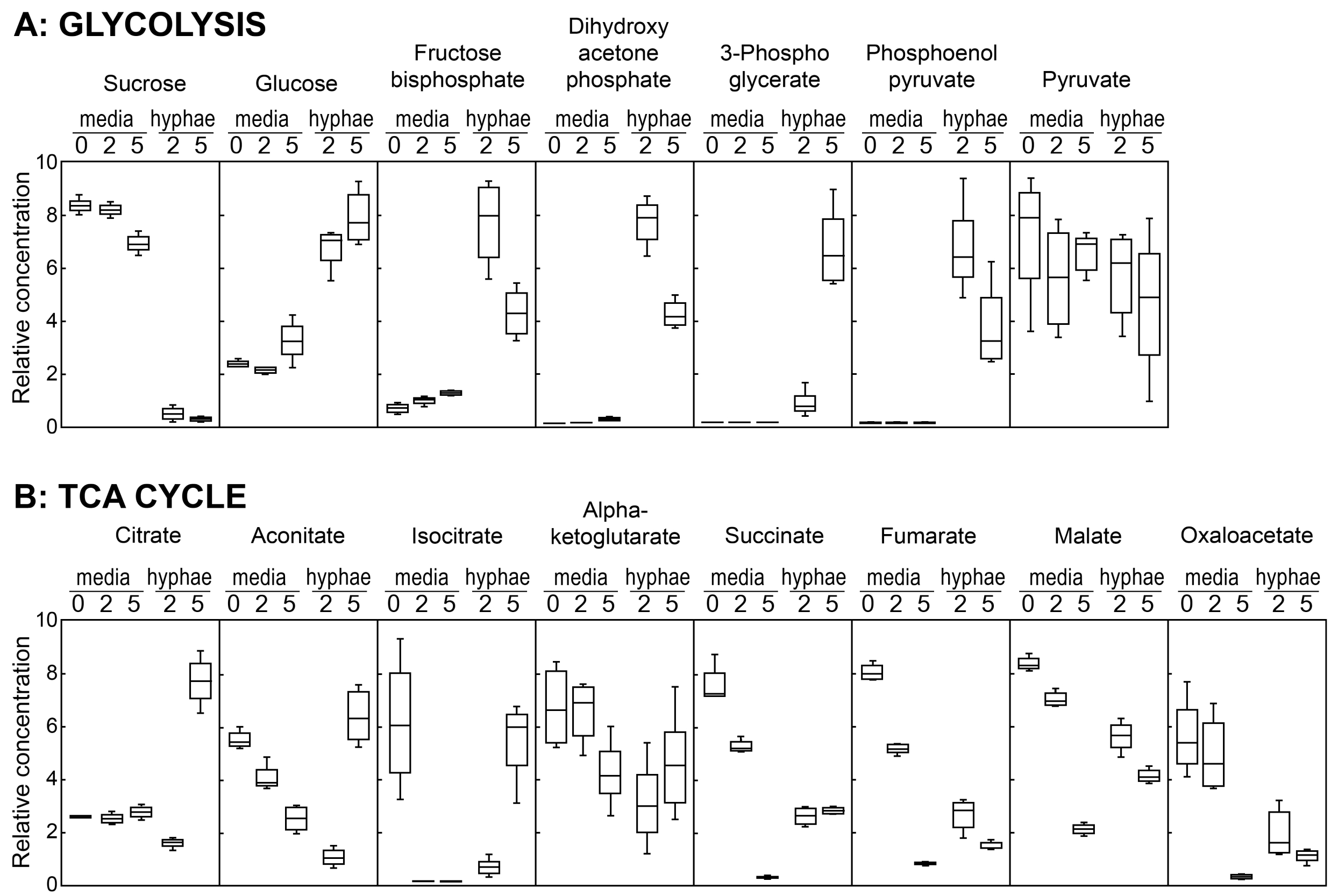

S1 Fig. Levels of carbon sources and metabolic intermediates in *P. infestans* hyphae and rye-sucrose media during the sporulation time course shown in Fig 2. **(A)** Sucrose, glucose, and glycolytic intermediates in media at days 0, 2, and 5 and in hyphae at days 2 and 5. **(B)** TCA cycle metabolites. The values are relative and should not be used to compare different compounds.
