# Supplementary material for: Transcriptional programs controlling sporulation in Phytophthora respond to nutrition, humidity, and cryptochrome-mediated light signals
Source: PLoS Pathog. 2026 Oct 12;22(10):e1014705. doi: 10.1371/journal.ppat.1014705 (PMC13674864; doi:10.1371/journal.ppat.1014705)
Supplement: Supplementary file 2 [file ppat.1014705.s010.pdf]

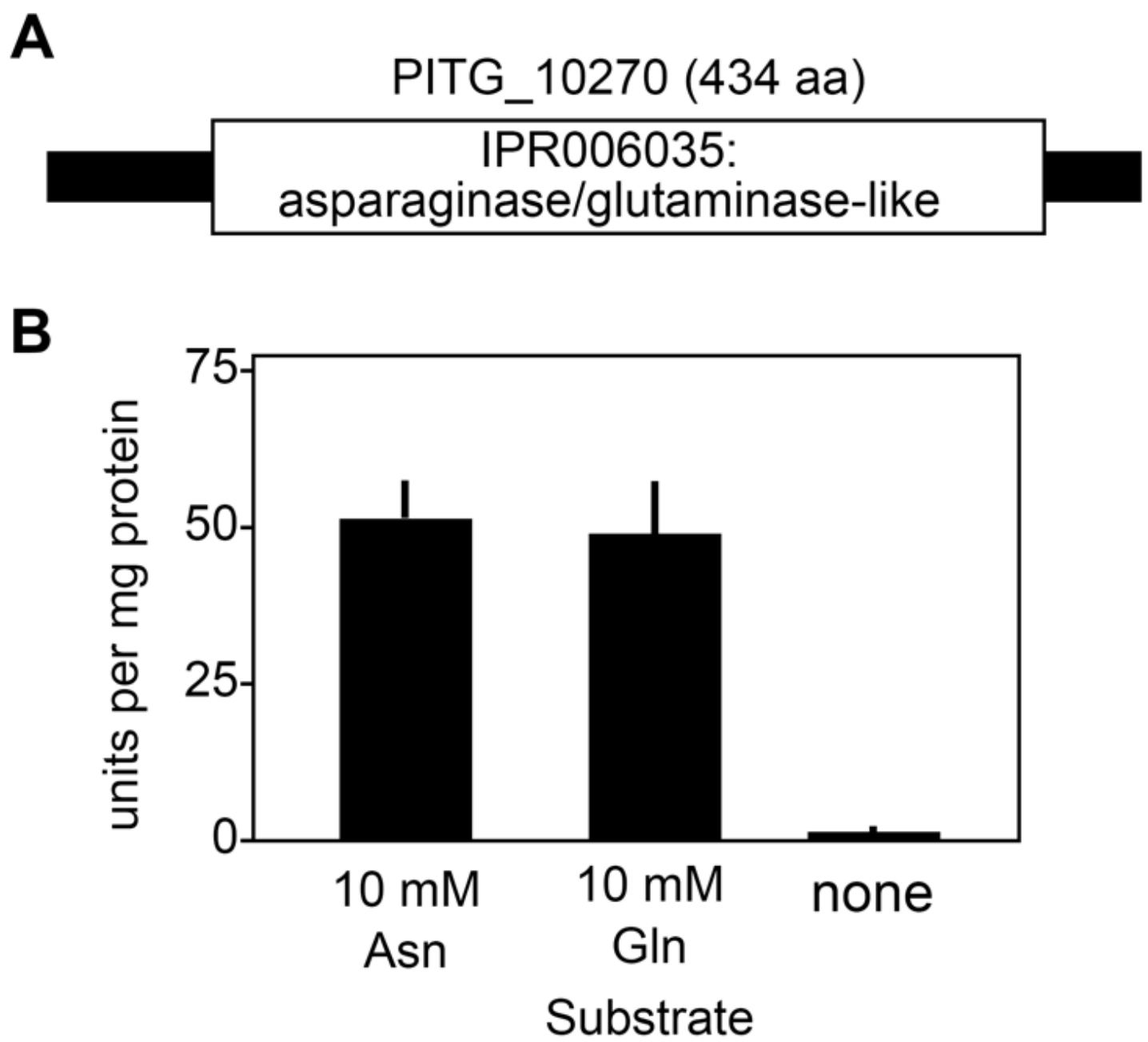

S2 Fig. Assay of *P. infestans* hyphal extracts for asparaginase-glutaminase activity. One unit is defined as the amount of enzyme that liberates 1 μmole of ammonia per minute at 21°C at pH 7.5.
