# Supplementary material for: Transcriptional programs controlling sporulation in Phytophthora respond to nutrition, humidity, and cryptochrome-mediated light signals
Source: PLoS Pathog. 2026 Oct 12;22(10):e1014705. doi: 10.1371/journal.ppat.1014705 (PMC13674864; doi:10.1371/journal.ppat.1014705)
Supplement: Supplementary file 3 [file ppat.1014705.s011.pdf]

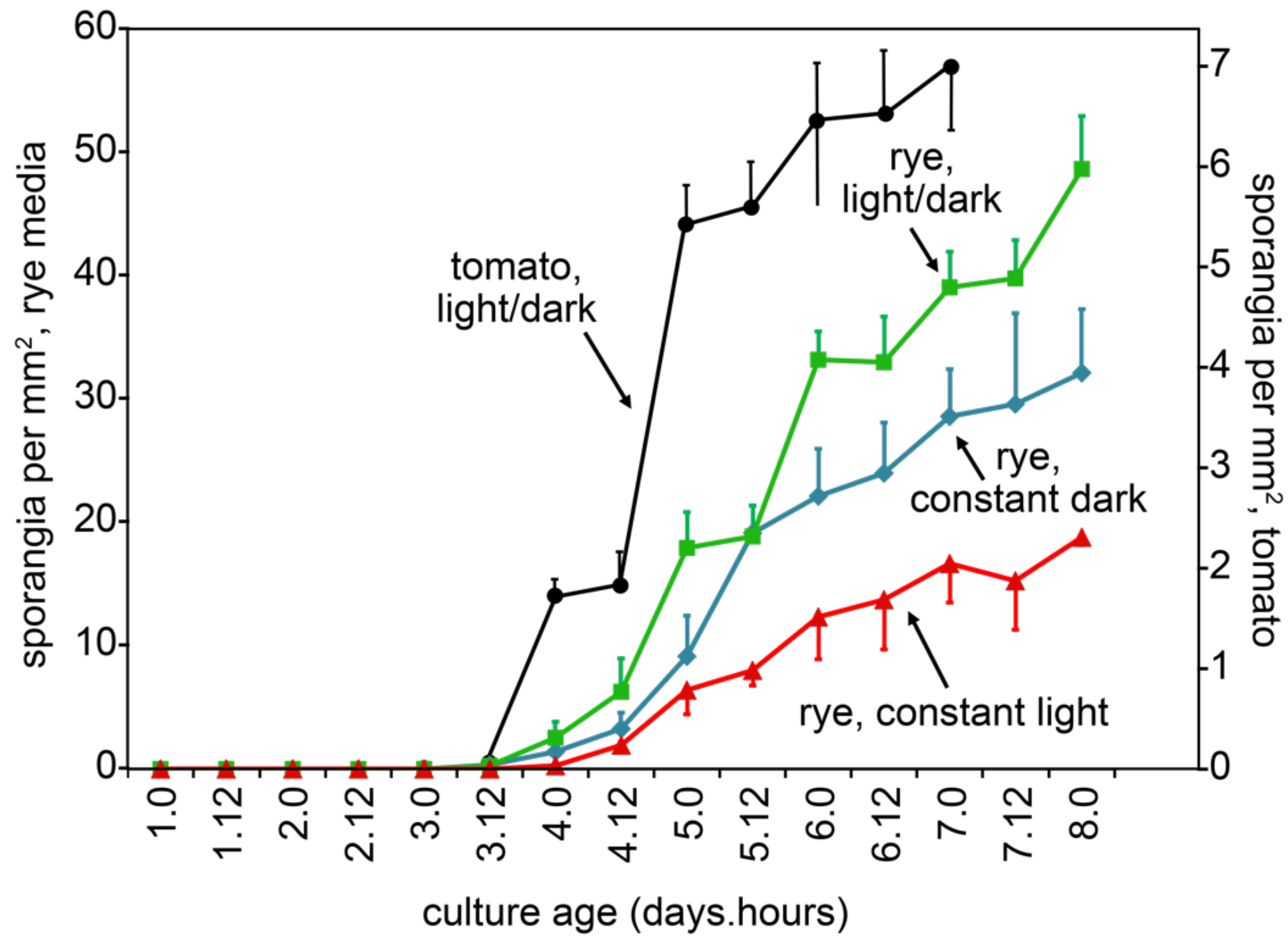

S3 Fig. Density of *P. infestans* sporangia in cultures grown in rye-sucrose media under darkness, constant light, a light/dark cycle, or in tomato leaflets grown in a light/dark sample. The values come from cultures or tissues grown in parallel to those used for RNA-seq and shown in Fig 4. The inhibitory effect of light was less than that shown in Fig 3 since its intensity was lower than what was used in the prior figure.
