# Supplementary material for: Transcriptional programs controlling sporulation in Phytophthora respond to nutrition, humidity, and cryptochrome-mediated light signals
Source: PLoS Pathog. 2026 Oct 12;22(10):e1014705. doi: 10.1371/journal.ppat.1014705 (PMC13674864; doi:10.1371/journal.ppat.1014705)
Supplement: Supplementary file 4 [file ppat.1014705.s012.pdf]

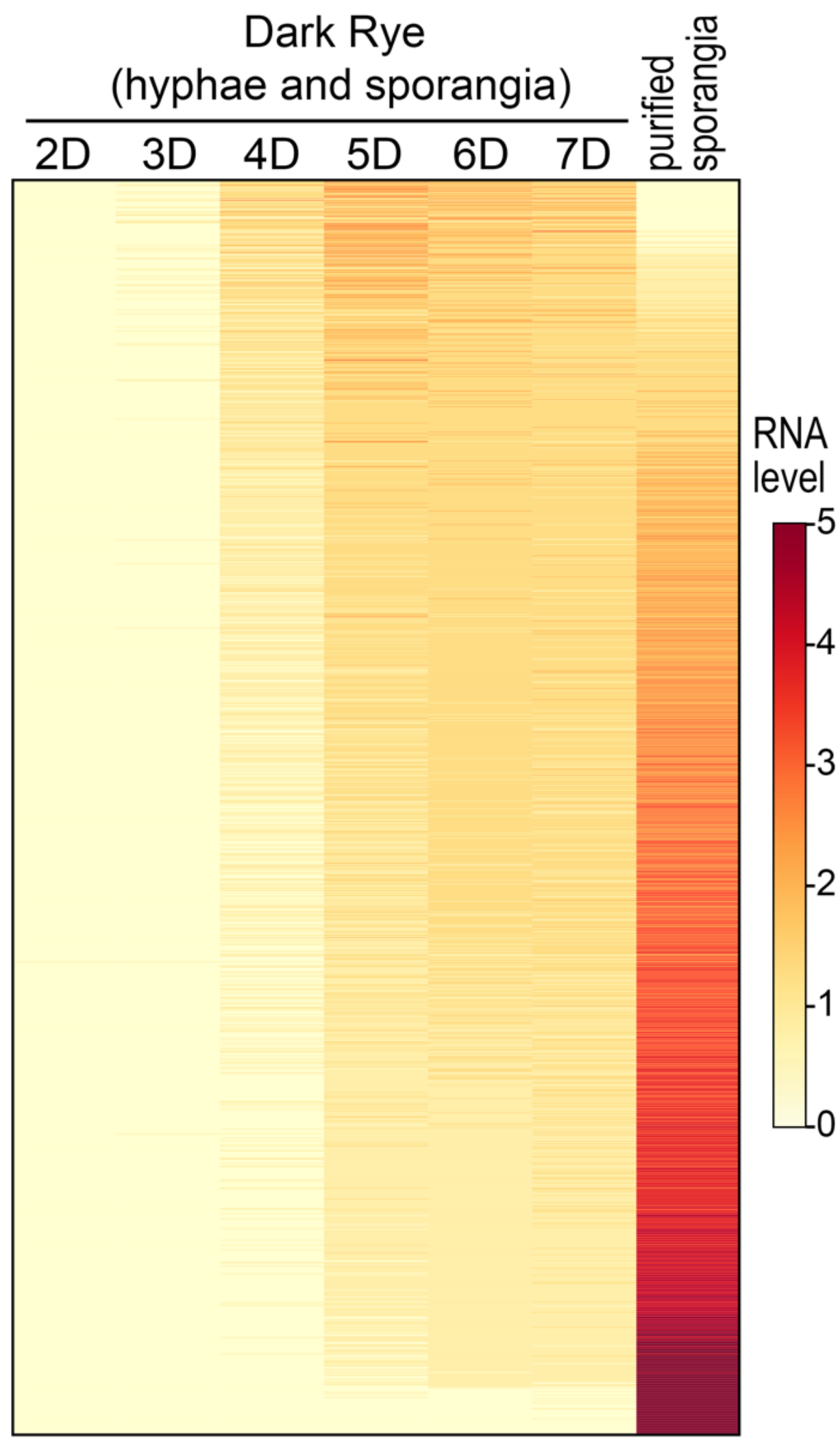

S4 Fig. Differential persistence of *P. infestans* mRNA in sporangia and sporulating hyphae. Shown are data from RNA-seq of sporangia purified from six-day cultures and hyphae (and sporangia when present) from days two to seven after inoculation. Data are normalized such that each row has a mean of 1.0. All tissues were grown in the dark.
