# Supplementary material for: Transcriptional programs controlling sporulation in Phytophthora respond to nutrition, humidity, and cryptochrome-mediated light signals
Source: PLoS Pathog. 2026 Oct 12;22(10):e1014705. doi: 10.1371/journal.ppat.1014705 (PMC13674864; doi:10.1371/journal.ppat.1014705)
Supplement: Supplementary file 5 [file ppat.1014705.s013.pdf]

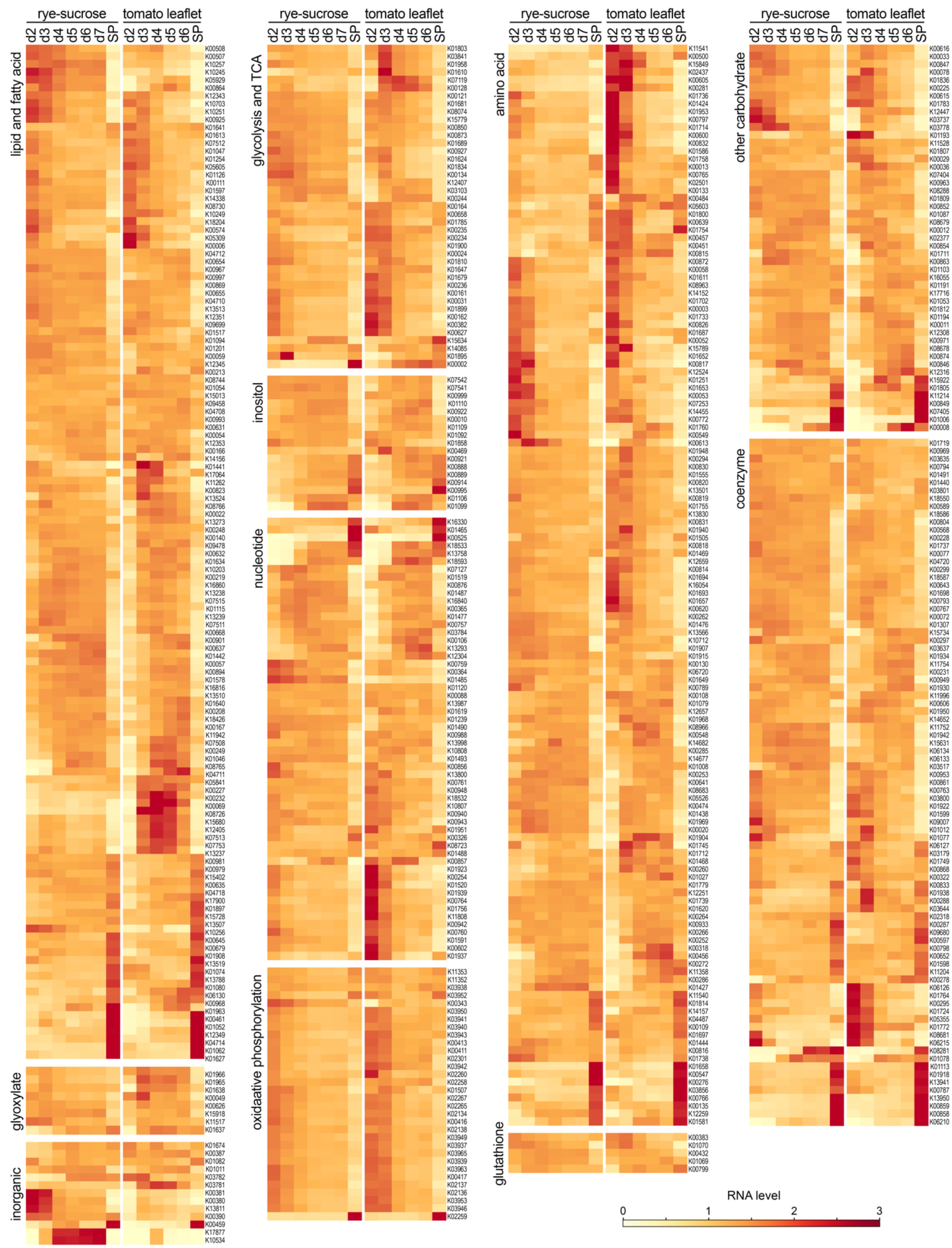

rye-sucrose
tomato leaflet
d2 d3 d4 d5 d6 d7 SP
d2 d3 d4 d5 d6 SP
lipid and fatty acid
glyoxylate
inorganic
glycolysis and TCA
inositol
nucleotide
oxidaative phosphorylation
amino acid
glutathione
other carbohydrate
coenzyme
RNA level
0
1
2
3

S5 Fig. mRNA levels of metabolic genes in time courses on rye-sucrose media (days 2 to 7 after inoculation) and tomato time courses (days 2 to 6), and sporangia (SP) harvested from those experiments on day 6. mRNA levels represented by the sum of FPKM values for each gene in the same KEGG orthology group, which are represented by the K numbers to the right of each heatmap. Data are normalized such that each row has a mean of 1.0.
