# Supplementary material for: Transcriptional programs controlling sporulation in Phytophthora respond to nutrition, humidity, and cryptochrome-mediated light signals
Source: PLoS Pathog. 2026 Oct 12;22(10):e1014705. doi: 10.1371/journal.ppat.1014705 (PMC13674864; doi:10.1371/journal.ppat.1014705)
Supplement: Supplementary file 6 [file ppat.1014705.s014.pdf]

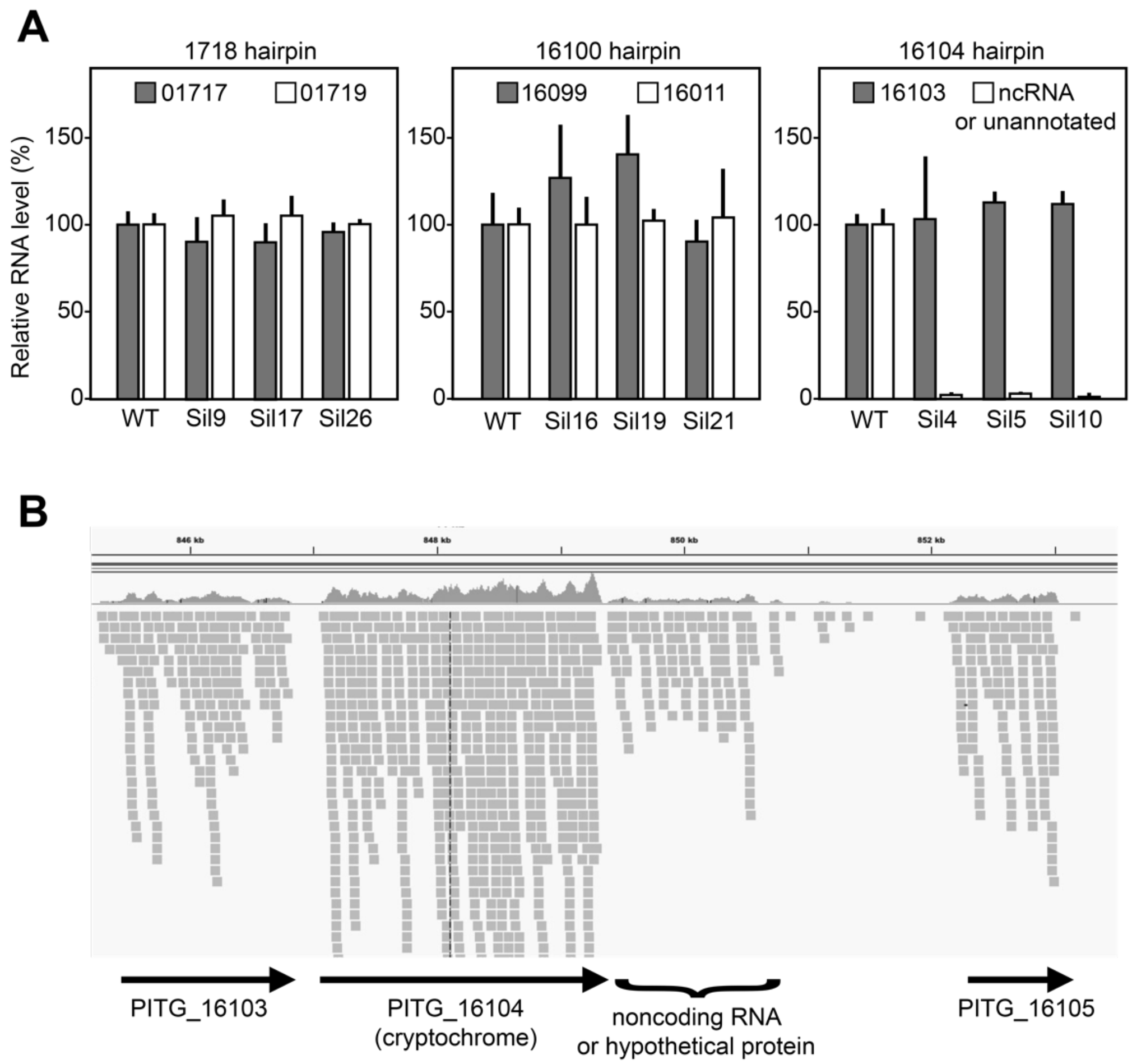

S6 Fig. Status of genes flanking the *P. infestans* cryptochromes in the knockdown transformants. **(A)** mRNA levels of genes flanking the three targeted loci, with expression determined by RT-qPCR and graphed relative to wild-type. PITG_01717 and PITG_01719 are left and right of PITG_01718, respectively, while PITG_16099 and PITG_16101 flank PITG_16100. With PITG_16104, the right flanking locus was not previously annotated and is labelled "new gene." **(B)** RNA-seq reads aligned to the region of the chromosome containing PITG_16104, pointing out the previously unannotated gene on its right. The three strains shown in Panel A failed to sporulate and exhibited silencing of both the cryptochrome and the new gene. Hence, the sporulation defect might be due to the silencing of either locus. The PITG_16104-silenced strains also showed poor growth, which could also be due to either locus. A subset of this data was reported previously.
