# Supplementary material for: Transcriptional programs controlling sporulation in Phytophthora respond to nutrition, humidity, and cryptochrome-mediated light signals
Source: PLoS Pathog. 2026 Oct 12;22(10):e1014705. doi: 10.1371/journal.ppat.1014705 (PMC13674864; doi:10.1371/journal.ppat.1014705)
Supplement: Supplementary file 7 [file ppat.1014705.s015.pdf]

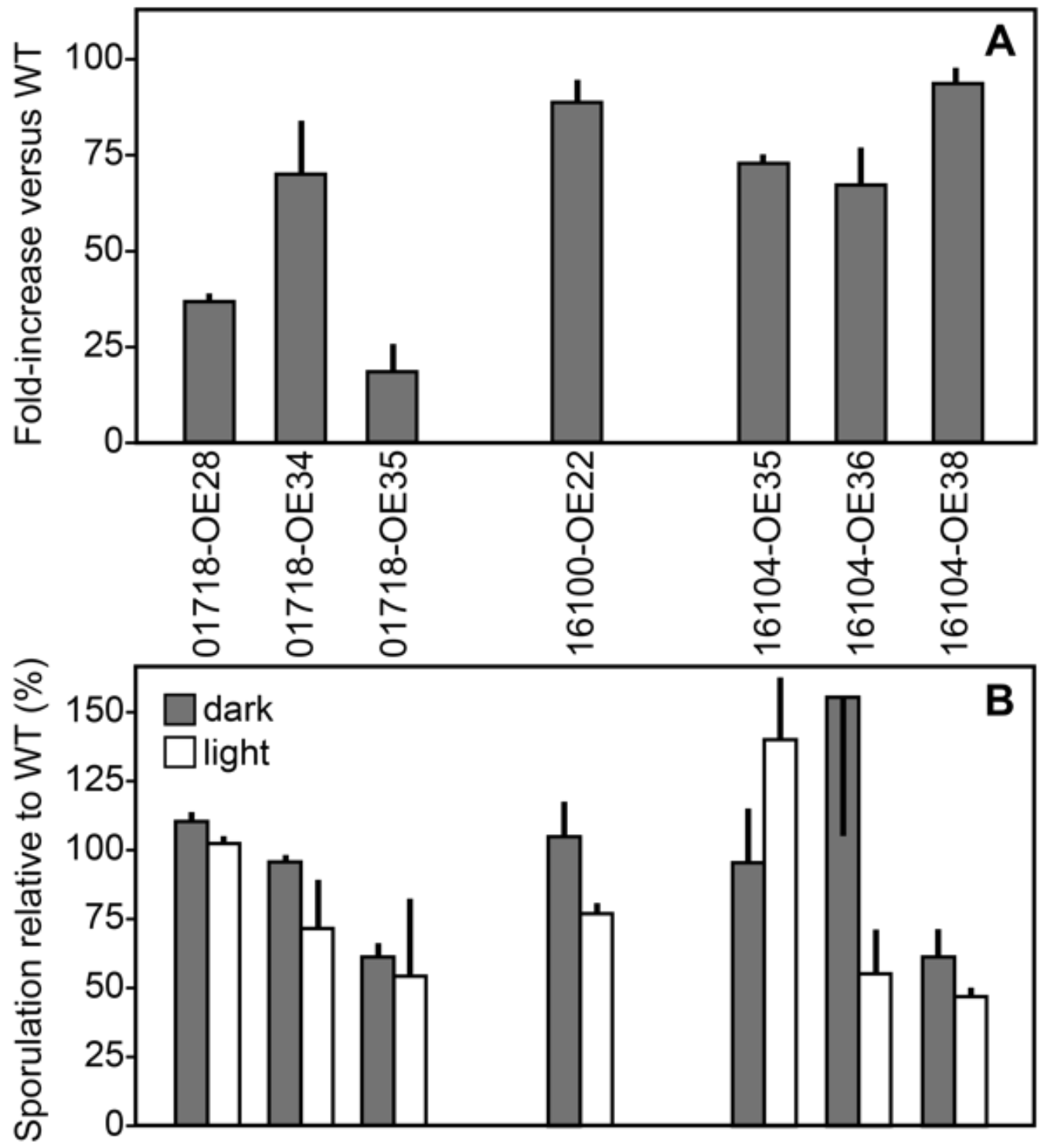

S7 Fig. Effect of overexpression of *P. infestans* cryptochromes on sporulation. **(A)** Fold-change increase in total cryptochrome mRNA levels in transformants relative to the wild-type progenitor based on RT-qPCR. Each protein (PITG_01718, 16100, and 16104) was expressed behind the strong *ham34* promoter with a FLAG tag. **(B)** Sporulation in transformants after six days growth under light or dark conditions. Values are expressed as percent relative to the wild-type progenitor strain 1306 and are the mean of three biological replicates from each of two experiments performed in different weeks.
