# Supplementary material for: Transcriptional programs controlling sporulation in Phytophthora respond to nutrition, humidity, and cryptochrome-mediated light signals
Source: PLoS Pathog. 2026 Oct 12;22(10):e1014705. doi: 10.1371/journal.ppat.1014705 (PMC13674864; doi:10.1371/journal.ppat.1014705)
Supplement: Supplementary file 8 [file ppat.1014705.s016.pdf]

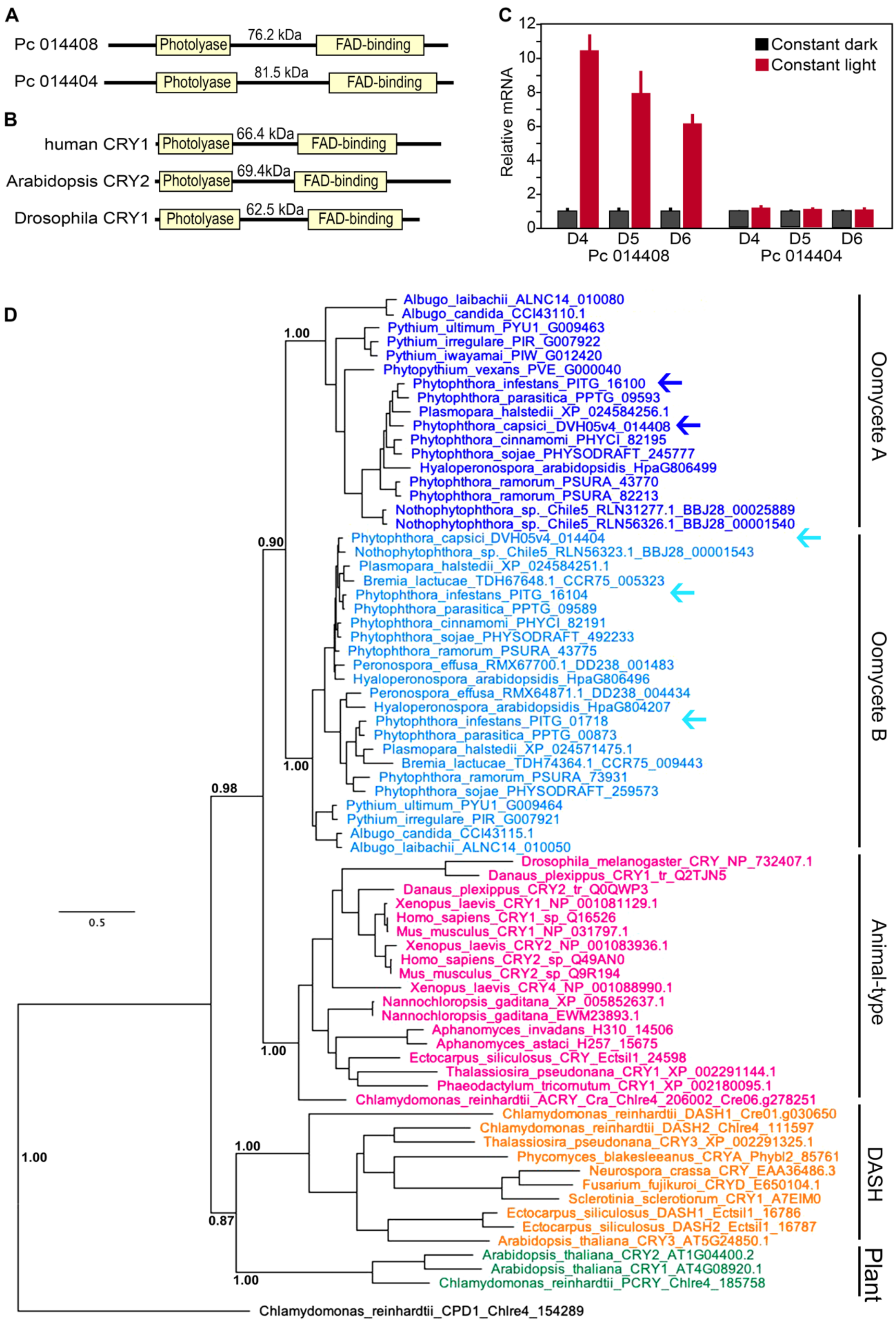

A
Pc 014408
Photolyase
76.2 kDa
FAD-binding
Pc 014404
Photolyase
81.5 kDa
FAD-binding
B
human CRY1
Photolyase
66.4 kDa
FAD-binding
Arabidopsis CRY2
Photolyase
69.4kDa
FAD-binding
Drosophila CRY1
Photolyase
62.5 kDa
FAD-binding
C
Relative mRNA
0
2
4
6
8
10
12
Constant dark
Constant light
D4
D5
D6
Pc 014408
D4
D5
D6
Pc 014404
D
Albugo_laibachii_ALNC14_010080
Albugo_candida_CCI43110.1
Pythium_ultimum_PYU1_G009463
Pythium_irregulare_PIR_G007922
Pythium_iwayamai_PIW_G012420
Phytopythium_vexans_PVE_G000040
Phytophthora_infestans_PITG_16100
Phytophthora_parasitica_PPTG_09593
Plasmopara_halstedii_XP_024584256.1
Phytophthora_capsici_DVH05v4_014408
Phytophthora_cinnamomi_PHYCI_82195
Phytophthora_sojae_PHYSODRAFT_245777
Hyaloperonospora_arabidopsidis_HpaG806499
Phytophthora_ramorum_PSURA_43770
Phytophthora_ramorum_PSURA_82213
Nothophytophthora_sp._Chile5_RLN31277.1_BBJ28_00025889
Nothophytophthora_sp._Chile5_RLN56326.1_BBJ28_00001540
Phytophthora_capsici_DVH05v4_014404
Nothophytophthora_sp._Chile5_RLN56323.1_BBJ28_00001543
Plasmopara_halstedii_XP_024584251.1
Bremia_lactucae_TDH67648.1_CCR75_005323
Phytophthora_infestans_PITG_16104
Phytophthora_parasitica_PPTG_09589
Phytophthora_cinnamomi_PHYCI_82191
Phytophthora_sojae_PHYSODRAFT_492233
Phytophthora_ramorum_PSURA_43775
Peronospora_effusa_RMX67700.1_DD238_001483
Hyaloperonospora_arabidopsidis_HpaG806496
Peronospora_effusa_RMX64871.1_DD238_004434
Hyaloperonospora_arabidopsidis_HpaG804207
Phytophthora_infestans_PITG_01718
Phytophthora_parasitica_PPTG_00873
Plasmopara_halstedii_XP_024571475.1
Bremia_lactucae_TDH74364.1_CCR75_009443
Phytophthora_ramorum_PSURA_73931
Phytophthora_sojae_PHYSODRAFT_259573
Pythium_ultimum_PYU1_G009464
Pythium_irregulare_PIR_G007921
Albugo_candida_CCI43115.1
Albugo_laibachii_ALNC14_010050
Drosophila_melanogaster_CRY_NP_732407.1
Danaus_plexippus_CRY1_tr_Q2TJN5
Danaus_plexippus_CRY2_tr_Q0QWP3
Xenopus_laevis_CRY1_NP_001081129.1
Homo_sapiens_CRY1_sp_Q16526
Mus_musculus_CRY1_NP_031797.1
Xenopus_laevis_CRY2_NP_001083936.1
Homo_sapiens_CRY2_sp_Q49AN0
Mus_musculus_CRY2_sp_Q9R194
Xenopus_laevis_CRY4_NP_001088990.1
Nannochloropsis_gaditana_XP_005852637.1
Nannochloropsis_gaditana_EWM23893.1
Aphanomyces_invadans_H310_14506
Aphanomyces_astaci_H257_15675
Ectocarpus_siliculosus_CRY_Ectsil1_24598
Thalassiosira_pseudonana_CRY1_XP_002291144.1
Phaeodactylum_tricornutum_CRY1_XP_002180095.1
Chlamydomonas_reinhardtii_ACRY_Cra_Chlre4_206002_Cre06.g278251
Chlamydomonas_reinhardtii_DASH1_Cre01.g030650
Chlamydomonas_reinhardtii_DASH2_Chlre4_111597
Thalassiosira_pseudonana_CRY3_XP_002291325.1
Phycomyces_blakesleeanus_CRYA_Phybl2_85761
Neurospora_crassa_CRY_EAA36486.3
Fusarium_fujikuroi_CRYD_E650104.1
Sclerotinia_sclerotiorum_CRY1_A7EIM0
Ectocarpus_siliculosus_DASH1_Ectsil1_16786
Ectocarpus_siliculosus_DASH2_Ectsil1_16787
Arabidopsis_thaliana_CRY3_AT5G24850.1
Arabidopsis_thaliana_CRY2_AT1G04400.2
Arabidopsis_thaliana_CRY1_AT4G08920.1
Chlamydomonas_reinhardtii_PCRY_Chlre4_185758
Chlamydomonas_reinhardtii_CPD1_Chlre4_154289
1.00
0.90
1.00
0.98
1.00
1.00
1.00
0.87
1.00
0.5
Oomycete A
Oomycete B
Animal-type
DASH
Plant

S8 Fig. Cryptochromes in *P. capsici* and other oomycetes. **(A)** structure of the two cryptochromes from *P. capsici.* **(B)** Cryptochromes from human, *Arabidopsis thaliana,* and *Drosophila melanogaster.* Comparison with panel A shows that the oomycete proteins lack the N-terminal extension found in the other species. **(C)** Expression of the *P. capsici* cryptochromes in cultures exposed to constant light or darkness, four to six days after inoculation. **(D)** Phylogenetic tree showing cryptochromes from oomycetes, animals, plants, and the related DASH proteins. The *P. capsici* and *P. infestans* proteins are marked with arrows. A more limited analysis of cryptochromes drew similar conclusions about how the oomycete proteins clustered [15]. While standard cryptochromes have been shown to regulate plant and animal development, and circadian clocks, CRY-DASH proteins repair UV-damaged single-stranded DNA. Both types of proteins are blue-light receptors.
